# A 20% nitrogen reduction threshold optimizes soil carbon stability and microbial functional resilience under long-term straw return in a rice–wheat rotation

**DOI:** 10.3389/fmicb.2026.1928083

**Published:** 2026-09-14

**Authors:** Guohan Si, Huanqi Jiang, Chenglin Peng, Dabing Xu, Shujun Zhao

**Affiliations:** 1 Institute of Plant Protection and Soil Fertilizer, Hubei Academy of Agricultural Sciences; Hongshan Field Scientific Observation and Research Station, Ministry of Agriculture and Rural Affairs, Wuhan, Hubei, China; 2 College of Life Science, Yangtze University, Jingzhou, Hubei, China

**Keywords:** aggregate stability, carbon management index, carbon sequestration, metagenomics, microbial carbon pump, nitrogen reduction, rice–wheat rotation, soil organic carbon

## Abstract

Reducing synthetic nitrogen (N) fertilizer while maintaining soil fertility and crop productivity is a major challenge for sustainable rice production. Straw return has been widely adopted to enhance soil organic carbon (SOC) sequestration; however, the mechanisms by which reduced N input interacts with straw return to regulate SOC stabilization remain poorly understood. Here we evaluated graded N reduction (0, 20, and 30%) under continuous straw return in a nine-year rice-wheat double-cropping experiment in central China. Soil carbon pools, aggregate stability, organic carbon functional groups, and microbial community structure and function were assessed using carbon fractionation, wet-sieving, FTIR spectroscopy, and metagenomic analyses. We found that 20% N reduction (80%NR) maintained grain yield comparable to conventional N fertilization while significantly increasing readily oxidizable organic carbon (ROOC, +18–26%), water-soluble organic carbon (WSOC, +12–21%), microbial biomass carbon (MBC, +15–24%), and carbon management index (CMI, +42–58% relative to NR). Moderate N reduction also enhanced macroaggregate-associated SOC and the relative abundance of aliphatic and aromatic carbon functional groups. Metagenomic analyses revealed that 80%NR enriched Firmicutes (+26%), Actinobacteria (+50%), and Nitrospira, reduced methanogenic archaea, and triggered a metabolic shift from CO oxidation (coxL/cutL, −15 to −22%) toward reductive carbon fixation via the Wood–Ljungdahl pathway (hdrA2, +6 to +19%). In contrast, 30% N reduction (70%NR) undermined subsurface aggregate stability and eroded microbial functional diversity, identifying 20% N reduction as an optimal threshold within the tested gradients. These findings provide a mechanistic basis for optimizing nitrogen management in sustainable rice production systems.

## Introduction

Soil organic carbon (SOC) is a fundamental determinant of soil fertility, crop productivity, and agroecosystem sustainability. Increasing SOC sequestration has become an important strategy for improving soil quality while mitigating climate change. In rice-based cropping systems, crop residue return represents one of the most effective approaches for enhancing SOC accumulation because it provides substantial organic carbon inputs and stimulates microbial-mediated nutrient cycling (Liu et al., 2022; Zhang et al., 2023). Long-term straw return has been shown to increase SOC content, improve aggregate stability, and enhance soil nutrient availability, thereby contributing to sustainable crop production (Xin et al., 2024). Despite these benefits, the efficiency of straw-derived carbon sequestration is strongly influenced by nitrogen (N) management. Nitrogen availability regulates straw decomposition, microbial growth, and carbon transformation processes, thereby affecting both the quantity and stability of SOC (Bei et al., 2022; Ren et al., 2024; Zhu et al., 2023). Recent metagenomic evidence further demonstrates that straw mulching combined with graded N inputs reshapes microbial functional potential—enriching carbon-degrading taxa and reductive fixation pathways that govern long-term carbon persistence (Yang H. et al., 2025). However, excessive N application may reduce microbial carbon-use efficiency and increase environmental risks, whereas insufficient N input may constrain microbial activity and crop productivity (Gaudel et al., 2024; Chen et al., 2026; Yang et al., 2026). Consequently, identifying an optimal balance between straw return and N reduction has become a key challenge for sustainable intensification of rice production systems.

Although numerous studies have evaluated the effects of straw return or nitrogen management individually, several critical knowledge gaps remain in paddy ecosystems (Sun et al., 2025; Liu et al., 2023; Song et al., 2025). First, nitrogen availability exerts contrasting effects on SOC dynamics: it can accelerate residue decomposition and extracellular enzyme production under moderate supply, yet suppress microbial carbon-use efficiency and promote soil acidification when excessive, while insufficient N triggers microbial N mining that alters plant-derived carbon inputs (Averill and Waring, 2018; Liu and Greaver, 2010). How these divergent processes interact with continuous straw return to shape the balance between labile and stable carbon fractions—and whether a threshold exists where reduced mineral N shifts this balance in flooded, anaerobic soils—remains unresolved. Second, the mechanisms responsible for SOC stabilization under reduced N input in paddy fields are unclear. Physical protection within aggregates and chemical stabilization of organic matter have rarely been evaluated simultaneously, and their interaction with methane-driven carbon loss during anaerobic straw decomposition remains unexplored. Third, while microbial communities are widely recognized as major drivers of soil carbon turnover, the extent to which microbial functional reorganization—specifically the trade-off between methanogenic carbon loss and reductive carbon fixation pathways—mediates SOC stabilization under graded N fertilization in flooded rice systems remains largely unresolved. Recent metagenomic evidence demonstrates that long-term straw mulching combined with graded N inputs reshapes microbial functional potential, enriching carbon-degrading taxa and reductive fixation pathways that govern long-term carbon persistence (Yang H. et al., 2025), yet how these functional shifts interact with aggregate protection and chemical recalcitrance under optimized N management is still unclear. Addressing these gaps is essential to identify an N reduction threshold that sustains productivity while minimizing carbon losses specific to paddy ecosystems.

We hypothesized that moderate nitrogen reduction under continuous straw return would maintain crop productivity while enhancing SOC stabilization through complementary mechanisms: (i) enriching carbon-degrading and nutrient-cycling microbiomes that transform straw-derived carbon into biologically active and necromass pools; (ii) promoting aggregate-mediated physical protection of organic carbon; (iii) enhancing the accumulation of chemically recalcitrant aliphatic and aromatic structures; and (iv) establishing a threshold-dependent response whereby 20% N reduction optimizes the balance between substrate acquisition and microbial biomass recycling, whereas deeper cuts (e.g., 30%) constrain anabolic capacity and subsurface aggregate stability, thereby weakening carbon persistence. This hypothesized ‘carbon-stabilizing metabolic state’ aligns with recent metagenomic evidence that straw–N interactions rewire microbial carbon-cycling networks toward functional configurations favoring long-term carbon persistence (Yang H. et al., 2025). To test these hypotheses, a 9-year field experiment with five straw-return and nitrogen-management treatments was conducted in a rice-based cropping system in central China. By integrating soil carbon fractionation, aggregate analysis, FTIR spectroscopy, and metagenomic sequencing, we aimed to (1) quantify threshold-dependent changes in SOC quantity and quality under graded N reduction, (2) evaluate the convergent contributions of aggregate protection, chemical recalcitrance, and microbial functional reprogramming to SOC persistence, and (3) identify the taxonomic and metabolic mechanisms that underpin a carbon-stabilizing metabolic state in paddy soils. Moving beyond correlative descriptions of straw return effects, this study provides a mechanistic basis—linking microbial necromass accumulation, aggregate-mediated physical protection, and reductive carbon fixation—for optimizing nitrogen management in sustainable rice production.

## Materials and methods

### Experimental design and plot management

This study was based on a long-term stationary field experiment that has been continuously operated for 9 years. Established in 2015 at the Songhe Experimental Station in Jingshan County, Hubei Province, central China, the experiment was carried out in a typical rice-wheat double-cropping system. The experimental site is located at 31°13′32.19″N, 113°15′32.42″E, at an elevation of 69 m above sea level. The region has a north subtropical humid monsoon climate, with a mean annual temperature of 16.6 °C and mean annual precipitation of 1,100 mm. The soil is classified as a homogeneous loamy fluvo-aquic paddy soil with a loamy texture. Prior to the initiation of the experiment in 2015, the topsoil (0–20 cm) had the following physicochemical properties: pH 6.79 (1:2.5 soil: water), soil organic matter 19.88 g kg^−1^, alkali-hydrolysable N 129.10 mg kg^−1^, available P 19.05 mg kg^−1^, available K 115.92 mg kg^−1^, and cation exchange capacity (CEC) 13.75 cmol kg^−1^. Since its establishment, identical experimental treatments, including diverse straw returning modes and gradient nitrogen fertilizer reduction regimes, have been consistently implemented in the same plots annually. Long-term and consistent treatment application enables progressive accumulation of treatment effects over time. The differences observed among treatments primarily reflect the inherent long-term ecological responses to specific management practices, which effectively eliminate or smooth the random disturbances induced by interannual climatic fluctuations (e.g., variations in precipitation and temperature anomalies) and other non-treatment confounding factors. The experiment is a randomized complete block design with three replicates and five treatments (15 plots, 30 m^2^ each). Plots are separated by 30-cm earthen ridges wrapped with plastic film to prevent lateral nutrient movement; a 0.5-m buffer strip surrounds each plot. Irrigation and drainage channels allow independent water management. Except for N rate and residue management, all agronomic practices (seeding density, transplanting spacing, pesticide and K/P input) followed local high-yield recommendations. The settings of five treatments and the nitrogen application amounts for each treatment are shown in Supplementary Table 1. Previous long-term field studies in rice–wheat cropping systems have demonstrated that straw incorporation combined with a 20–30% reduction in mineral nitrogen (N) input constitutes a feasible and safe management range for sustaining crop yield (Chen K. et al., 2023). In contrast, N reduction exceeding 30% often triggers early-season soil N immobilization and consequently leads to yield losses. Accordingly, the 20 and 30% N reduction levels were adopted in the present study.

Straw return management. Both rice and wheat straw were returned to the field annually under the straw-return treatments. Wheat straw was chopped to <10 cm, incorporated to a depth of 15–20 cm at a rate of 6,000 kg ha^−1^ (dry weight) in late May, with a moisture content of 35%, C/N ratio of 95.8, total C of 420.5 g kg^−1^, total N of 4.39 g kg^−1^, total P of 0.58 g kg^−1^, and total K of 19.28 g kg^−1^. Rice straw was chopped to <15 cm, incorporated to a depth of 15–20 cm at a rate of 10,000 kg ha^−1^ (dry weight) in early October, with a moisture content of 65%, C/N ratio of 61.4, total C of 444.8 g kg^−1^, total N of 7.24 g kg^−1^, total P of 0.96 g kg^−1^, and total K of 23.97 g kg^−1^. Straw-free treatments (N0R0 and NR0) had all aboveground residue removed from the plots. Fertilizer type, application rate and straw return amount for each treatment are presented in Supplementary Table 1.

### Soil sampling and treatment

Intact soil samples were collected on 8 October 2024. Within each plot, five subsamples were taken along an “S” pattern from 0–10 cm and 10–20 cm horizons. After removal of visible roots and debris, samples were pooled per layer, placed in polyethene bags, transported on ice to the laboratory within 2 h, and gently sieved (<2 mm). Each composite sample was split into three portions: (i) field-moist (4 °C, 48 h) for dissolved organic C (DOC) and microbial biomass C (MBC); (ii) air-dried for aggregate fractionation, total organic C (TOC) and Fourier-transform infrared (FTIR) spectroscopy; (iii) metagenomic analyses.

Soil carbon fractionation and carbon pool index analysis.

Total organic carbon (TOC) was determined by the Walkley–Black dichromate oxidation method with external heating (Nelson and Sommers, 1982). Briefly, 0.5 g of air-dried soil (passed through a 0.25-mm sieve) was digested with 5 mL of 0.5 M K₂Cr₂O₇ and 5 mL of concentrated H₂SO₄ at 150–160 °C for 5 min. The residual dichromate was titrated with 0.25 M FeSO₄ using o-phenanthroline as an indicator, and TOC was calculated from the difference in FeSO₄ consumption between the sample and a blank (Walkley and Black, 1934).

Microbial biomass carbon (MBC) was measured by the chloroform fumigation–extraction method (Vance et al., 1987). Fresh soil was split into two subsamples: one was fumigated with ethanol-free CHCl₃ in a vacuum desiccator for 24 h to lyse microbial cells, and the other was left unfumigated as a control. Both subsamples were extracted with 0.5 M K₂SO₄ (soil-to-solution ratio 1:4) for 30 min and filtered. Organic carbon in the extracts was determined using a TOC analyzer. MBC was calculated as the difference in extractable organic carbon between fumigated and unfumigated soils, divided by a conversion factor of 0.45 (Vance et al., 1987).

Particulate organic carbon (POC) was isolated by physical fractionation. Air-dried soil (passed through a 2-mm sieve) was dispersed in 5 g L^−1^ sodium hexametaphosphate solution and shaken overnight. The suspension was then wet-sieved through a 53-μm sieve; the >53 μm fraction was collected as the particulate fraction, oven-dried at 60 °C, ground, and analyzed for organic carbon by the Walkley–Black method.

Readily oxidizable organic carbon (ROOC) was determined by permanganate oxidation (Blair et al., 1995). Air-dried soil (1.5 g, <0.25 mm) was shaken with 25 mL of 333 mmol L^−1^ KMnO₄ for 1 h, centrifuged at 2,000 rpm for 5 min, and the supernatant was diluted 1:250 with deionised water. Absorbance was measured at 565 nm, and ROOC was calculated from the decrease in KMnO₄ concentration, assuming that 1 mmol KMnO₄ oxidises 9 mg of carbon.

Water-soluble organic carbon (WSOC) was extracted by shaking 10 g of air-dried soil (<2 mm) with 20 mL of deionised water (soil-to-water ratio 1:2) at 25 °C for 30 min. The suspension was filtered through a 0.45-μm membrane, and WSOC in the filtrate was measured directly using a TOC analyzer.

Carbon pool indices were calculated following Blair et al. (1995), using the adjacent long-term uncultivated soil as the reference. Readily oxidizable organic carbon (ROOC) was designated as the active organic carbon (AOC) pool. The indices were computed as follows:


Carbon pool index(CPI)=sampleTOC/referenceTOC

Non−active organic carbon(NAOC)=TOC−AOC

Carbon pool activity(CPA)=AOC/NAOC

Carbon pool activity index(CPAI)=sampleCPA/referenceCPA

Carbon management index(CMI)=CPI×CPAI×100

Wet-sieving was employed to determine the particle-size distribution and stability of soil aggregates (Ni et al., 2024). A DM2000 soil aggregate analyzer (Beijing Mengchuang Weiye Technology Co., Ltd., Beijing, China) was used to sequentially pass the soil samples through sieves with mesh sizes of 2, 1, 0.5, 0.25, and 0.053 mm, separating six fractions of water-stable aggregates: >2, 1–2, 0.5–1, 0.25–0.5, 0.053–0.25 mm, and <0.053 mm. After separation, the aggregate fractions were oven-dried at 60 °C, weighed, ground to pass a 100-mesh sieve, and then analyzed for organic carbon content.

Fourier-transform infrared (FTIR) spectroscopy was performed on a Nicolet Summit X spectrometer (Thermo Fisher Scientific, Waltham, MA, USA). Bulk soil samples were scanned over the full mid-infrared range of 4,000–400 cm^−1^. For quantitative functional-group analysis, raw spectra were subjected to peak deconvolution using Peakfit v4.12 software (Systat Software, San Jose, CA, USA). Each spectrum was fitted with Gaussian line-shape functions to resolve overlapping absorption bands, and the integrated areas of sub-peaks were quantified within three diagnostic wavenumber regions: O-alkyl C (C–O stretching, centered at ~1,031 cm^−1^), alkyl C (aliphatic C–H stretching, 3,000–2,800 cm^−1^), and aromatic C (C=C stretching, ~1,635 cm^−1^). Relative abundance (%) = (integrated area of target functional group / sum of total integrated areas of three diagnostic regions) × 100%. The calculated relative-abundance values for each treatment and soil layer are summarized in Supplementary Table 5.

### Metagenomic library construction and bioinformatics for *de novo* assembly, functional annotation, and taxonomic profiling of microbial communities

Following the qualification of genomic DNA, the samples were mechanically sheared to generate fragment libraries. Fragmented DNA was subjected to end-repair, 3′-dA tailing, adapter ligation, size selection, and PCR enrichment to produce sequencing libraries. Libraries passing quality control (QC) were sequenced on the Illumina platform. Raw reads were quality-filtered to yield high-quality clean reads for downstream bioinformatic analyses. Clean reads were assembled *de novo*, open reading frames (ORFs) were predicted, and a non-redundant (NR) gene catalogue was constructed. Summary statistics of gene sequences, including the number of genes and sequence length, are presented in Supplementary Table 2. Functional annotation was performed against public (e.g., KEGG) and specialized (e.g., CAZy) databases, and taxonomic profiling was conducted to determine species composition and relative abundance. Raw reads frequently contain low-quality or artefactual sequences; rigorous filtering is therefore required before analysis. The filtering pipeline consisted of: 1. Adapter trimming and quality filtering of raw tags using fastp v0.23.2 (Open Bioinformatics Foundation) (Chen et al., 2018) to generate clean tags. 2. Host contamination removal by alignment of clean tags to the host reference genome with Bowtie 2 v2.4.4 (Johns Hopkins University, Baltimore, MD, USA) (Langmead and Salzberg, 2012). All raw sequencing data reported in this study have been deposited in the NCBI Sequence Read Archive (SRA) under BioProject accession number PRJNA1392060.

Metagenomic assembly was performed using MEGAHIT v1.2.9 (The University of Hong Kong, Hong Kong, China) (Li et al., 2015); contigs shorter than 300 bp were discarded. Assembly quality was evaluated with QUAST v5.2.0 (Centre for Algorithmic Biotechnology, St. Petersburg, Russia) (Gurevich et al., 2013). Gene prediction on assembled contigs was performed using MetaGeneMark v3.26 (Georgia Institute of Technology, Atlanta, GA, USA) with default parameters (Zhu et al., 2010) (http://exon.gatech.edu/meta_gmhmmp.cgi, v3.26) with default parameters. Construction of a non-redundant gene set via CD-HIT v4.8.1 (University of California, San Diego, CA, USA) (Steinegger and Söding, 2017) (protein identity ≥ 90%, coverage ≥ 80%). Functional annotation by homology searches against the KEGG database (Release 102.0, Kanehisa Laboratories, Kyoto, Japan) (Kanehisa, 2004) and CAZy database (Release 8.0, AFMB, Marseille, France).

### Statistical analysis

One-way analysis of variance (one-way ANOVA) followed by Tukey’s honestly significant difference (HSD) *post-hoc* test was used to compare treatment effects on soil properties, yield parameters, carbon fractions, aggregate stability indices, and microbial alpha-diversity indices. Significance was set at *p* < 0.05. All statistical analyses were performed using SPSS (version 26.0, IBM Corp., Armonk, NY, USA) and R (version 4.3.1, R Core Team). For metagenomic functional gene comparisons, the Mann–Whitney *U* test with Benjamini–Hochberg false discovery rate (FDR) correction was applied (FDR < 0.05) to account for multiple testing.

## Results

### Straw return maintains rice yield under 20% nitrogen reduction

Straw return effectively compensated for the reduction in mineral nitrogen input and maintained rice productivity across nitrogen-reduction treatments (Figure 1). The no-fertilizer and no-straw control (N0R0) recorded the lowest 1,000-grain weight (TSW, 19.27 g) and grain yield (9,270 kg ha^−1^). Application of 100% mineral N without straw (NR0) increased TSW to 21.73 g and yield to 10,920 kg ha^−1^, but reduced the number of grains per ear to 145 and maintained a harvest index (HI) of 56.6%. Returning rice straw at full N (NR) preserved TSW (22.09 g) and HI (56.6%), yet marginally decreased yield to 10,635 kg ha^−1^; this reduction was not statistically significant compared with NR0 (*p* > 0.05), indicating a transient immobilization effect without agronomic penalty. A 20% N reduction coupled with straw return (80%NR) further elevated TSW to 23.54 g while sustaining grains per ear (142), HI (56.1%) and yield (10,635 kg ha^−1^); yield and HI did not differ significantly from those under NR (*p* > 0.05), corroborating that a widened C/N ratio did not compromise grain filling. Deeper N reduction (70%NR) maximized TSW (24.92 g) and HI (57.7%) and restored yield to 10,830 kg ha^−1^, which was statistically comparable to both NR0 and NR (*p* > 0.05) (Figure 1). Consequently, straw return buffered yield loss under 20–30% N reduction, and the 70% N regime even numerically out-yielded the conventional N-plus-straw treatment without reaching statistical significance (*p* > 0.05).

**Figure 1:**
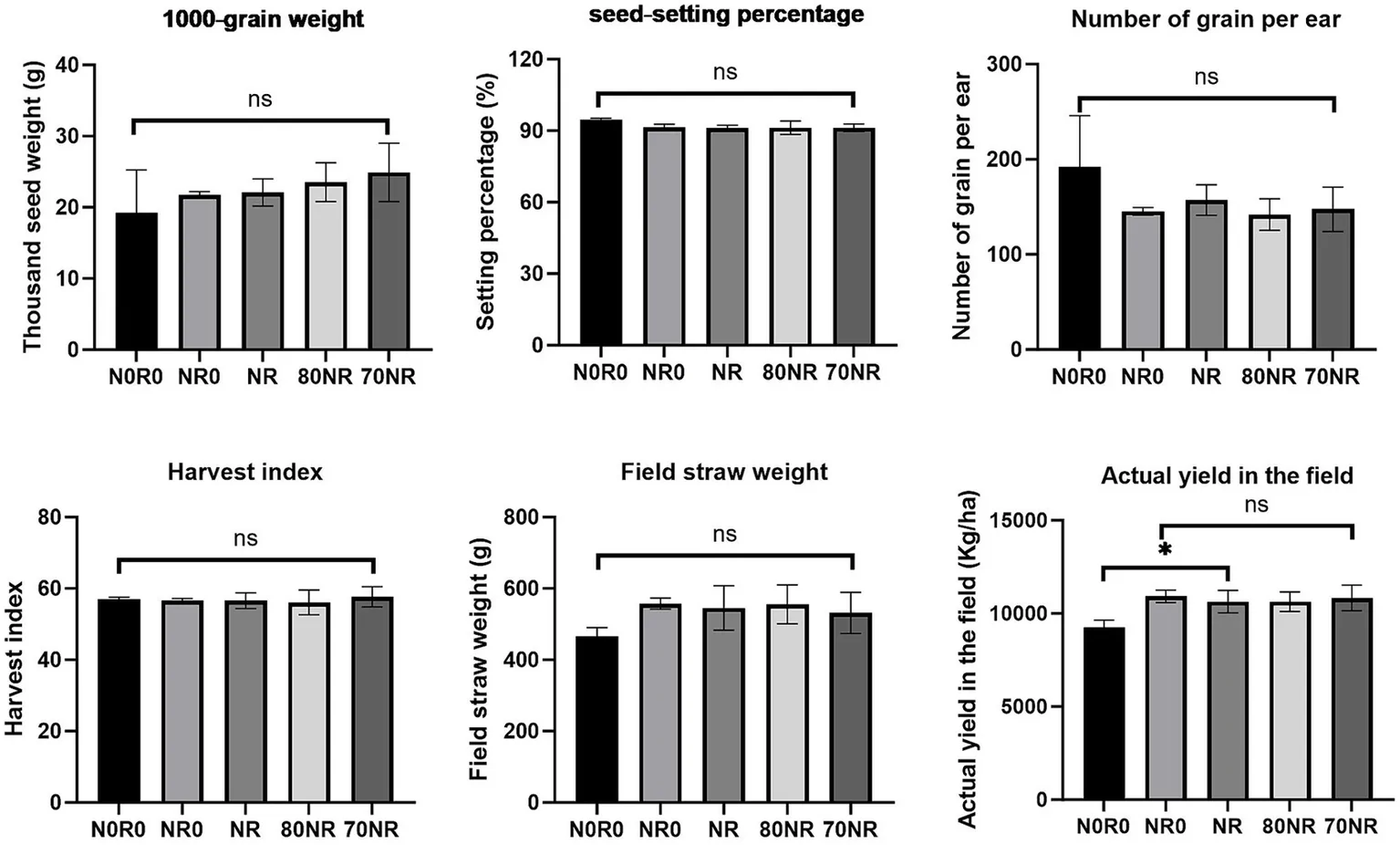
Agronomic performance of rice under combined straw return and graded nitrogen reduction. Bars represent 1,000-grain weight: The weight of 1,000 filled grains randomly sampled from each plot and oven-dried to constant weight; seed-setting percentage (%): The ratio of filled grains to total spikelets per panicle, determined from 20 randomly selected panicles per plot; grains per panicle (number of grains per ear): The total number of spikelets (filled and unfilled) per panicle, counted from 20 randomly selected panicles per plot; harvest index (%): The ratio of grain yield to total aboveground biomass (grain + straw); grain-to-straw rate: The ratio of grain dry weight to straw dry weight; field straw weight: The dry weight of aboveground plant residue after grain removal; and actual yield in the field (kg ha^−1^): The actual grain yield harvested from each plot and converted to kilograms per hectare. Values are means ± SD (*n* = 3). Error bars indicate standard deviation; ns, not significant (*p* > 0.05); *, significant at *p* < 0.05.

Meanwhile, Nitrogen agronomic efficiency (AE_N) followed the gradient NR (11.15 kg kg^−1^) ≈ NR0 (11.30 kg kg^−1^) < 80%NR (12.78 kg kg^−1^) < 70%NR (14.22 kg kg^−1^) (Supplementary Table 3). The 20% N reduction increased AE_N by 13.2% relative to NR while maintaining statistically comparable grain yield, indicating improved nitrogen-use efficiency without agronomic penalty. Notably, 70%NR further elevated AE_N to 14.22 kg kg^−1^, but this gain was accompanied by declining subsurface aggregate stability and microbial functional diversity, underscoring the ecological trade-off beyond the 20% threshold. These results suggest that long-term straw return enhanced nutrient cycling and improved nutrient-use efficiency, thereby buffering potential yield penalties associated with reduced mineral N inputs. The maintenance of grain yield under 80%NR highlights the potential for reducing fertilizer dependency while sustaining agricultural productivity.

### Moderate nitrogen reduction increased biologically active carbon pools while maintaining total SOC

In the 0–10 cm layer, total organic C (TOC) followed the gradient N0R0 (15.92 g kg^−1^) < 70%NR (16.31 g kg^−1^) ≈ 80%NR (16.62 g kg^−1^) < NR (16.80 g kg^−1^), with straw-containing treatments being significantly higher than straw-free controls (*p* < 0.05) (Figure 2). Particulate organic carbon (POC) remained comparatively stable across treatments (5.38–5.72 g kg^−1^), whereas readily oxidizable organic carbon (ROOC) increased markedly with straw addition, peaking at 6.53 g kg^−1^ under 80%NR—36% higher than N0R0 (Figure 2). Water-soluble organic carbon (WSOC) exhibited a similar trend, reaching 13.97 mg kg^−1^ under 70%NR. Microbial biomass carbon (MBC) showed the greatest sensitivity, increasing from 168 mg kg^−1^ in N0R0 to 230 mg kg^−1^ in 80%NR. This 37% enhancement indicates that a 20% reduction in mineral N under continuous straw return maximized microbial anabolism without compromising TOC accumulation.

**Figure 2:**
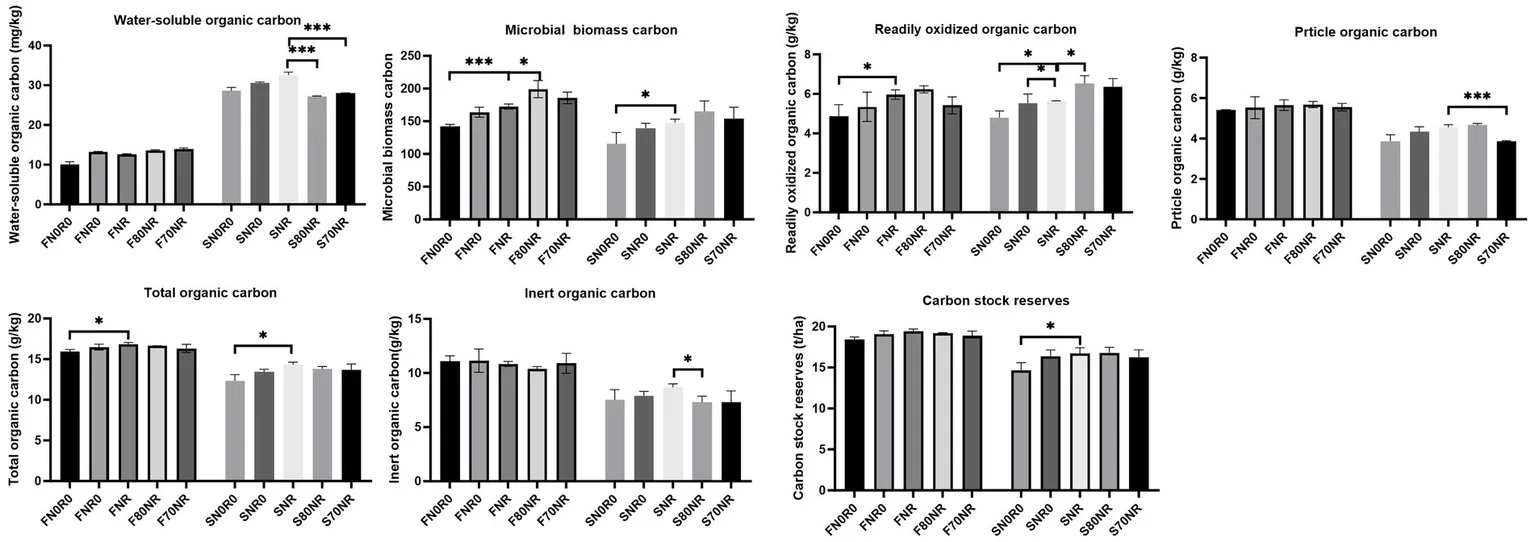
Total organic carbon (TOC), particulate organic carbon (POC), readily oxidizable organic carbon (ROOC), water-soluble organic carbon (WSOC), and microbial biomass carbon (MBC) under straw return and reduced nitrogen fertilization at 0–10 cm and 10–20 cm soil depths (mean ± SD, *n* = 3). ns, not significant (*p* > 0.05); *, significant at *p* < 0.05. **p < 0.01, ***p < 0.001.

At 10–20 cm, the stimulatory effect of straw return persisted. TOC increased from 12.32 g kg^−1^ (N0R0) to 14.32 g kg^−1^ (NR) and remained at or above 13.65 g kg^−1^ under both reduced-N treatments (80%NR and 70%NR) (Figure 2). POC and ROOC again peaked under 80%NR (4.68 and 6.23 g kg^−1^, respectively), while WSOC showed a slight, non-significant decrease with reduced N inputs. MBC followed the same ranking as in the topsoil, with 80%NR sustaining 169 mg kg^−1^—significantly higher than NR (150 mg kg^−1^) and the straw-free controls (124–158 mg kg^−1^). Collectively, these results demonstrate that straw return elevates both total and labile C throughout the 0–20 cm soil profile; reducing mineral N by up to 30% within this straw-retained system does not diminish TOC and can even amplify ROOC and MBC, thereby optimizing the quantity and biological availability of the soil organic carbon pool.

Furthermore, MAOC, calculated as SOC minus POC, exhibited significant treatment-dependent variations in both F (0–10 cm) and S (10–20 cm) soil layers (*p* < 0.01) (Supplementary Figure 2). By contrast, the POC/SOC (%) ratio showed no significant treatment effects across F and S layers (*p* > 0.05) (Supplementary Figure 2). Soil C/N ratios were altered by straw return and graded N reduction (Supplementary Table 4). In the 0–10 cm layer, the C/N ratio followed the gradient N0R0 (8.79) < NR (9.02) < NR0 (9.10) < 70%NR (9.31) < 80%NR (9.40), indicating that moderate N reduction under straw return widened the soil C/N stoichiometry. At 10–20 cm, the C/N ratio was more responsive to deeper N cuts: 70%NR recorded the highest value (9.67), exceeding NR (9.27) by 4.3% and N0R0 (9.17) by 5.5%. The elevated C/N ratio under 80%NR coincided with increased microbial biomass carbon and carbon management index, suggesting that this stoichiometric shift favored microbial carbon retention over respiration. In contrast, the further widening of C/N under 70%NR in the subsurface aligned with reduced aggregate stability and microbial functional diversity, indicating that excessive C/N imbalance may constrain microbial anabolic capacity.

### Changes in labile carbon pools under graded nitrogen reduction with straw return

At 0–10 cm, N0R0 exhibited the lowest Carbon Pool Index (CPI, 2.05) and Carbon Management Index (CMI, 102), with a Carbon Pool Activity (CPA) of merely 0.43. NR0 increased CPI to 2.62 and CMI to 186, whereas straw return at full N (NR) further elevated CPI to 2.93 and maintained CMI at 198. Reducing mineral N to 80% of the recommended rate (80%NR) did not reduce CPI (2.75) but significantly enhanced CPA and the carbon pool activity index (CPAI) to 0.65 and 0.76, respectively, yielding the highest CMI (218) among all treatments (Figure 3). Further reduction to 70%NR marginally decreased CPI to 2.44 yet preserved high CPA (0.65) and a CMI of 189, confirming that a 20–30% decrease in mineral N under continuous straw return optimizes surface-layer carbon lability without compromising total carbon stock (Figure 3).

**Figure 3:**
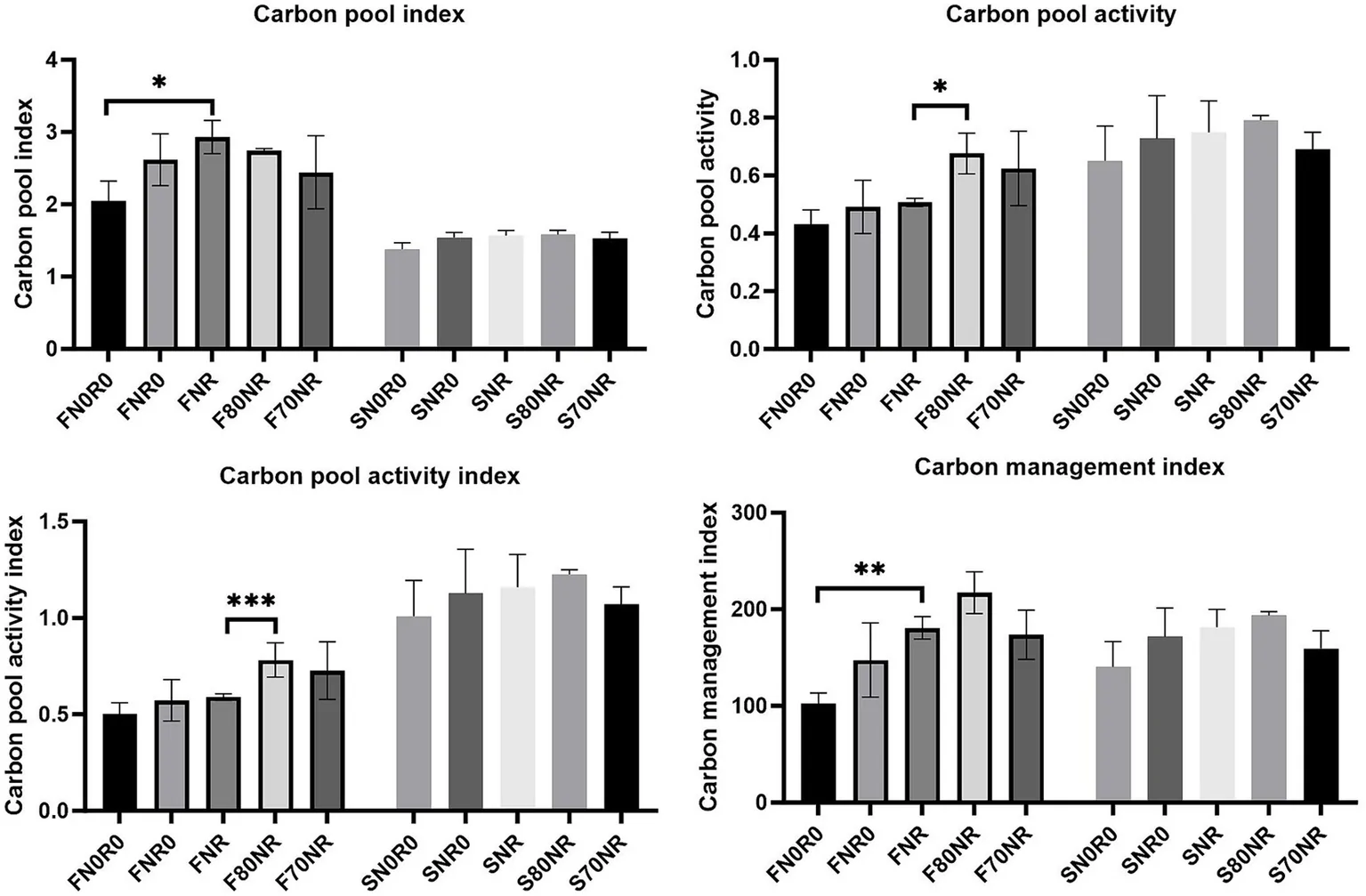
Carbon pool indices under different straw and nitrogen management practices at 0–10 cm (F) and 10–20 cm (S) soil depths. Carbon pool index (CPI), carbon pool activity (CPA), carbon pool activity index (CPAI), carbon management index (CMI). Values are means ± SD (*n* = 3). Error bars indicate standard deviation. *p < 0.05, **p < 0.01, ***p < 0.001.

At 10–20 cm, analogous trends were observed. N0R0 again recorded the lowest CPI (1.38) and CMI (112) (Figure 3). Full N plus straw (NR) elevated CPI to 1.57 and CMI to 172. Consistent with the topsoil, 80%NR achieved the greatest CPA (0.79) and CPAI (1.23), driving CMI to a profile maximum of 194 (Figure 3). The 70%NR treatment showed a marginal decline in CPA (0.69) and CMI (159) but remained significantly above NR0 and N0R0. Throughout the 0–20 cm profile, non-active organic carbon varied only minimally (7.85–11.18 g kg^−1^), indicating that improvements in CMI were governed primarily by expansion of the active fraction rather than by changes in the recalcitrant pool.

### Straw return enhances surface-soil aggregate stability under moderate N reduction, but a 30% N cut weakens subsurface structure in a rice rotation

In the 0–10 cm layer, R0.25 increased progressively from 20 (N0R0) to 31 under full straw plus recommended N (NR) (Figure 4); NR simultaneously recorded the greatest mean weight diameter (MWD, 1.24 mm) and geometric mean diameter (GMD, 0.46 mm) and the lowest fractal dimension (D, 2.48) (Figure 4), indicating superior aggregate stability. Moderate N reduction (80%NR) maintained R0.25 at 29 and MWD at 1.14 mm, values statistically comparable to those under NR (*p* > 0.05), whereas a deeper N cut (70%NR) slightly decreased R0.25 to 28 and MWD to 1.12 mm, yet both remained above the straw-free controls. The fractal dimension increased to 2.56–2.58 under reduced-N treatments, reflecting a modest decline in aggregate hierarchy as the C/N ratio widened (Figure 4).

**Figure 4:**
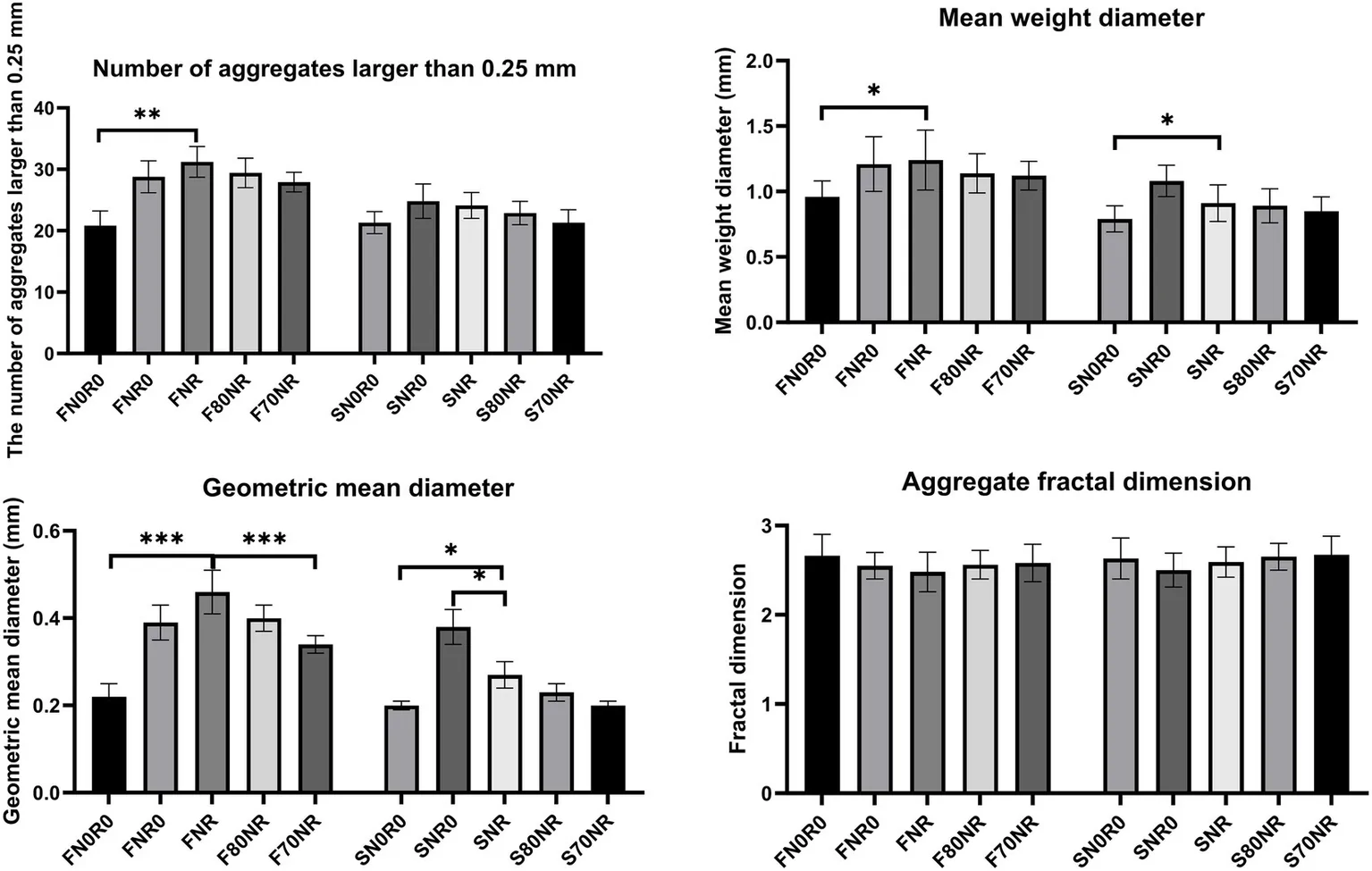
Water-stable aggregate stability indices under different straw and nitrogen management practices at 0–10 cm (F) and 10–20 cm (S) soil depths. Panels show R₀.₂₅: number of aggregates >0.25 mm; MWD: mean weight diameter (mm); GMD: geometric mean diameter (mm); and D: fractal dimension. Values are means ± SD (*n* = 3). *p < 0.05, **p < 0.01, ***p < 0.001.

In the 10–20 cm layer, straw effects were less pronounced but followed analogous trends. NR maximized R0.25 (24), MWD (0.91 mm) and GMD (0.27 mm) and minimized D (2.50). Both reduced-N treatments (80%NR and 70%NR) showed lower R0.25 (23 and 21) and MWD (0.89 and 0.85 mm) relative to NR, with D increasing to 2.65–2.67, implying that subsurface aggregation is more sensitive to diminished N input. Overall, continuous straw incorporation enhanced water-stable macro-aggregation and structural stability in the rice-season paddy; reducing mineral N by 20% preserved these benefits, whereas a 30% N cut began to weaken aggregate resilience, particularly in the subsurface horizon (Figure 4).

### Straw return with a 20% N reduction promotes carbon occlusion in macroaggregates, while deeper N cuts attenuate subsurface reallocation

In the 0–10 cm layer, both N0R0 and NR0 were dominated by microaggregates; the <0.053 mm and 0.053–0.25 mm fractions jointly accounted for approximately 54% of SOC. Straw incorporation shifted this distribution: the macroaggregate (>2 mm) carbon contribution increased from 23–24 to 27%, while the <0.053 mm fraction decreased to 24.6%. Under 80%NR, the macroaggregate share was maintained at 27%, while the 0.25–0.5 mm fraction peaked at 15.1% and the silt-clay (<0.053 mm) contribution declined to 18.4%, indicating enhanced carbon association within coarse mesoaggregates. Further reduction to 70%NR maximized the macroaggregate carbon contribution to 29.6% and reduced the finest fraction to 14.7%, corroborating that a wider C/N ratio promotes carbon sequestration in structurally stable, large aggregates (Figure 5).

**Figure 5:**
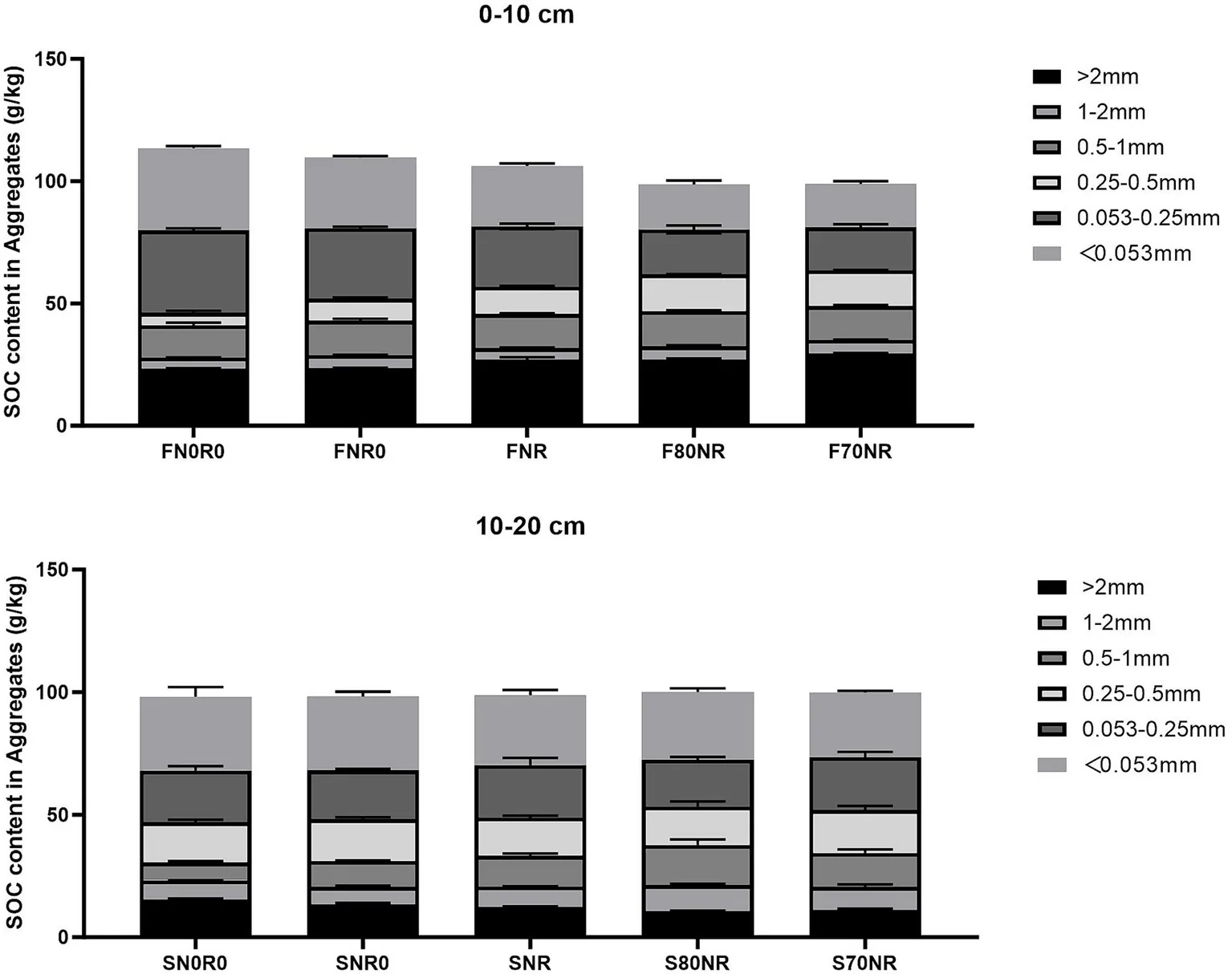
Soil organic carbon content in water-stable aggregate fractions under different straw and nitrogen management practices at 0–10 cm and 10–20 cm soil depths. Treatments: N0R0 (no fertilizer, no straw), NR0 (conventional N without straw), NR (conventional N with straw), 80%NR (20% N reduction with straw), and 70%NR (30% N reduction with straw). Soil samples were separated into six water-stable aggregate-size fractions (>2, 1–2, 0.5–1, 0.25–0.5, 0.053–0.25, and <0.053 mm) by wet-sieving using a DM2000 soil aggregate analyzer.

In the 10–20 cm layer, subsurface aggregates responded weakly; macroaggregate carbon contributions remained ≤13% across all treatments. Nevertheless, 80%NR elevated the 0.5–1 mm fraction to 16.3% (compared with 10.6% under NR0) and reduced the <0.053 mm share to 27.6%. A 30% N reduction (70%NR) attenuated these gains, returning the 0.5–1 mm contribution to 13.9% and the finest fraction to 26.3%. Collectively, straw return reallocates organic carbon from silt-clay-sized particles into macro- and mesoaggregates; this beneficial redistribution is maximized when mineral N is reduced by 20%, whereas deeper N cuts partially offset the improvement in the subsurface horizon (Figure 5).

### Quantitative FTIR peak-area analysis reveals a compositional shift toward recalcitrant carbon structures under moderate N reduction

FTIR spectroscopy combined with quantitative peak-area integration revealed significant shifts in soil organic matter functional-group composition following straw return and graded nitrogen reduction (Figure 6; Supplementary Figure 1; Supplementary Table 5). In the 0–10 cm layer, the O-alkyl C proportion, representing labile polysaccharides, was lowest under 80%NR (9.83%), decreasing by 4.7% relative to N0R0 (10.31%) and by 15.5% relative to NR0 (11.63%). Concurrently, the aromatic C=C proportion under 80%NR reached 1.98%, exceeding N0R0 (1.80%) by 10.0% and NR (1.70%) by 16.5%, and representing the highest value among straw-return treatments. The aliphatic C–H proportion under 80%NR (1.07%) was comparable to NR (1.02%) but lower than NR0 (1.52%) and 70%NR (1.45%).

**Figure 6:**
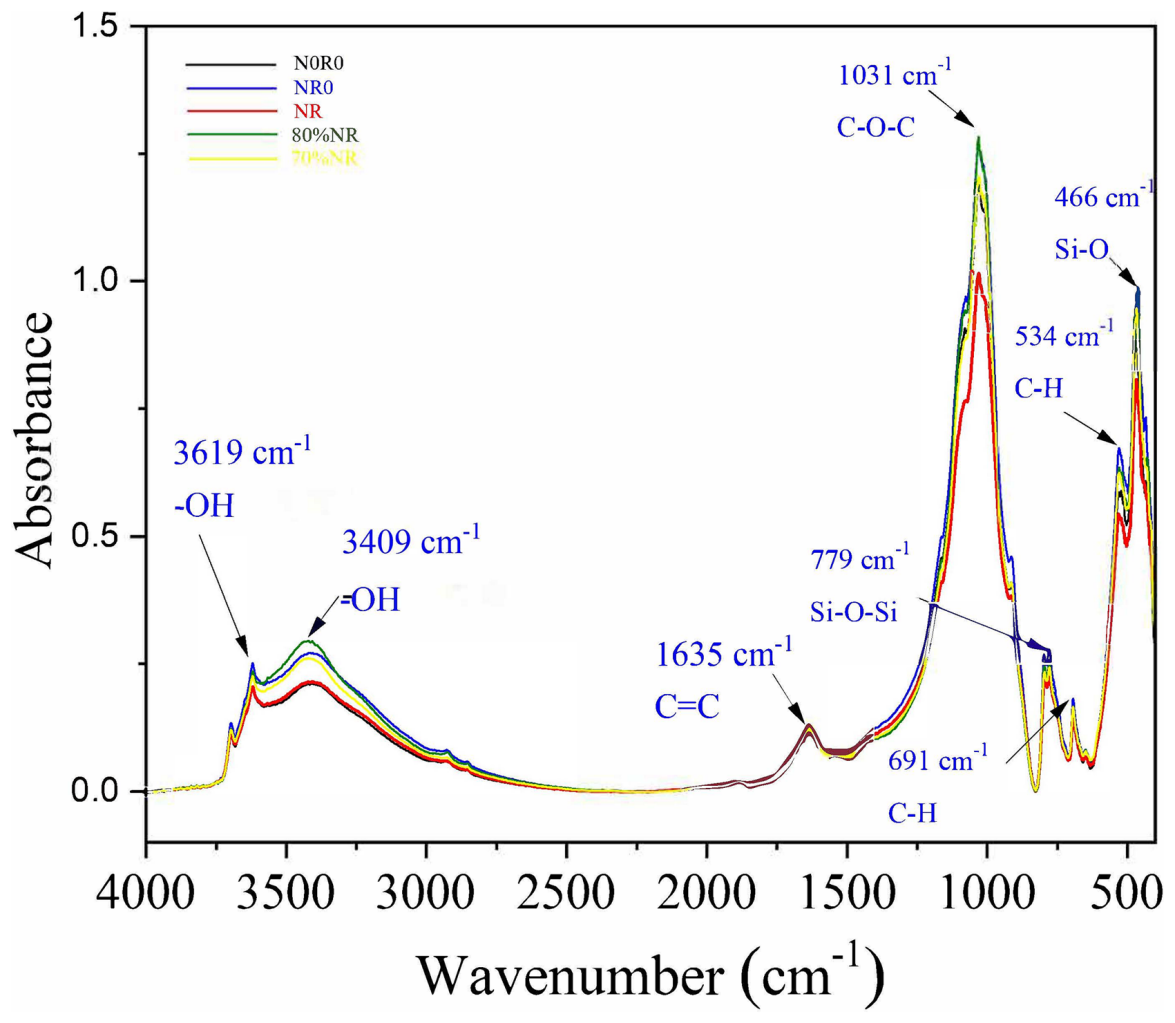
Fourier-transform infrared (FTIR) spectra of bulk paddy soil under combined straw return and graded nitrogen reduction. Spectra were acquired on a Nicolet Summit X spectrometer (Thermo Fisher Scientific, Waltham, MA, USA) over the mid-infrared range of 4,000–500 cm^−1^. Treatments: N0R0 (no fertilizer, no straw), NR0 (conventional N without straw), NR (conventional N with straw), 80%NR (20% N reduction with straw), and 70%NR (30% N reduction with straw). Key absorption bands are annotated: O–H stretching (3,619 and 3,409 cm^−1^), aromatic C=C stretching (~1,635 cm^−1^), O-alkyl C–O stretching (~1,031 cm^−1^), Si–O–Si bending (~779 cm^−1^), aliphatic C–H bending (~691 and 534 cm^−1^), and Si–O bending (~466 cm^−1^).

At 10–20 cm, the aromatic C=C proportion under 80%NR (2.20%) exceeded N0R0 (1.73%) by 27.2% and NR (1.93%) by 14.0%, approaching the level of NR0 (2.34%). The O-alkyl C proportion in 80%NR (9.96%) was lower than in NR (11.36%) and NR0 (10.73%). Notably, the aliphatic C–H proportion under 80%NR (1.01%) exceeded that under NR (0.80%) by 26.3%, indicating a relative enrichment of aliphatic structures under moderate N reduction in the subsurface.

Collectively, these quantitative FTIR data demonstrate that 80%NR promoted a compositional shift toward aromatic and aliphatic structures—particularly in the 10–20 cm layer—while constraining the accumulation of labile O-alkyl compounds. This redistribution from oxygen-containing polysaccharides toward more biochemically recalcitrant carbon forms supports enhanced long-term chemical stabilization of soil organic matter under moderate nitrogen reduction.

### Moderate nitrogen reduction reshaped microbial communities toward carbon-acquiring taxa

N reduction under straw return induced substantial taxonomic restructuring across both soil depths (Figure 7). At the phylum level (Figure 7A), Proteobacteria dominated (27–31%), followed by Chloroflexi (15–18%) and Acidobacteria (10–13%). Successive N reductions restructured the microbiome (Figure 7C): Firmicutes increased from 4.2% (N0R0) to 5.3% (70%NR), and Actinobacteria rose from 6.8 to 10.2%, while Proteobacteria declined from 31 to 27%, Acidobacteria from 13 to 10%, and Chloroflexi from 18 to 15%. At the genus level (Figures 7B,D), 80%NR maximized Sphingomonas (3.7%) and Nitrospira (0.62%), whereas methanogenic archaea decreased by 18–24% relative to NR. Streptomyces, Paenibacillus and Chitinophaga collectively increased by 28% under 80%NR compared with NR0. The 10–20 cm layer showed a parallel trajectory, with Firmicutes and Actinobacteria increasing by 3.1 and 2.8%, respectively, under 80%NR; however, 70%NR was associated with reduced functional diversity, as Shannon diversity dropped by 8.4% relative to 80%NR in the subsurface.

**Figure 7:**
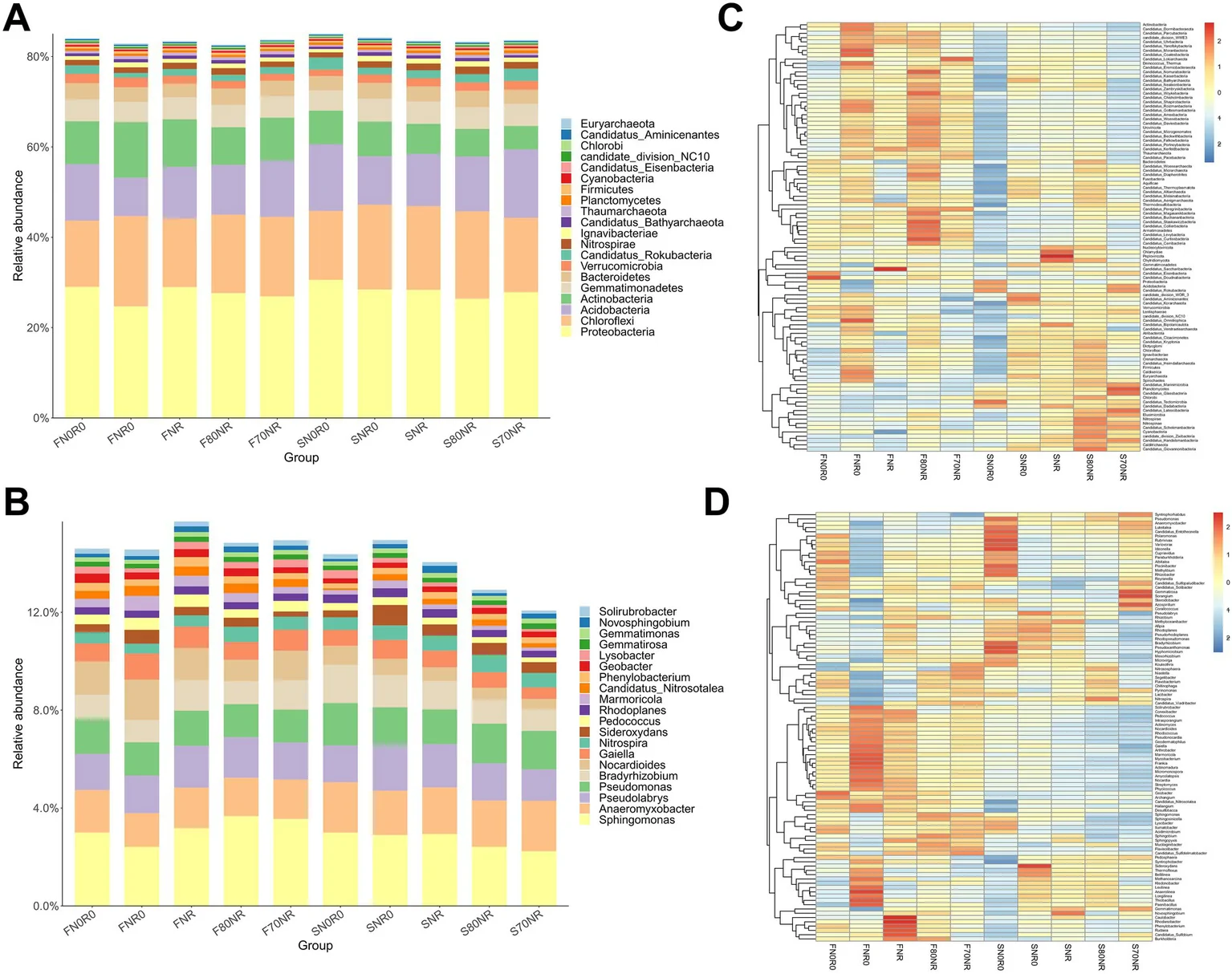
Taxonomic composition of soil microbial communities at the phylum and genus levels under combined straw return and graded nitrogen reduction. Treatments: N0R0 (no fertilizer, no straw), NR0 (conventional N without straw), NR (conventional N with straw), 80%NR (20% N reduction with straw), and 70%NR (30% N reduction with straw). Prefixes F and S denote the 0–10 cm and 10–20 cm soil layers, respectively. **(A)** Relative abundance of the top 20 phyla across treatments. **(B)** Relative abundance of the top 20 genera across treatments. **(C)** Heatmap showing the abundance distribution of phyla across individual samples (row *Z*-score normalized, hierarchical clustering by Ward’s method). **(D)** Heatmap showing the abundance distribution of genera across individual samples (row Z-score normalized, hierarchical clustering by Ward’s method). Color intensity in **(C,D)** indicates the *Z*-score of relative abundance (red, high; blue, low). Data are derived from metagenomic taxonomic profiling (*n* = 3 per treatment).

### CAZy and KEGG functional profiles under graded N reduction

GT1, CBM50, GH13, GH5 and GH23 dominated the CAZy profile (Figure 8A). Under 80%NR, GH5 (endo-cellulase) and GH43 (arabinoxylanase/β-xylosidase) peaked at 0.047 and 0.098, respectively, representing 15.6 and 22.3% increases over NR0 (Figure 8B). CBM48 and CBM50 increased linearly with N reduction, rising by 12.4 and 18.7% under 80%NR relative to NR, whereas GH23 and GT1 declined by 9.8 and 7.2%. At 10–20 cm, GH5 and CBM50 rose under reduced N, accompanied by >30% increases in GH18 (chitinase) and GH25 (lysozyme) compared with NR0. KEGG profiling (Figures 8C,D) revealed that carbohydrate metabolism (0.142‰) and energy metabolism (0.138‰) remained stable (<0.1‰ variation) across N-reduction treatments, while membrane transport increased by 0.08‰ and translation by 0.06‰ under 80%NR.

**Figure 8:**
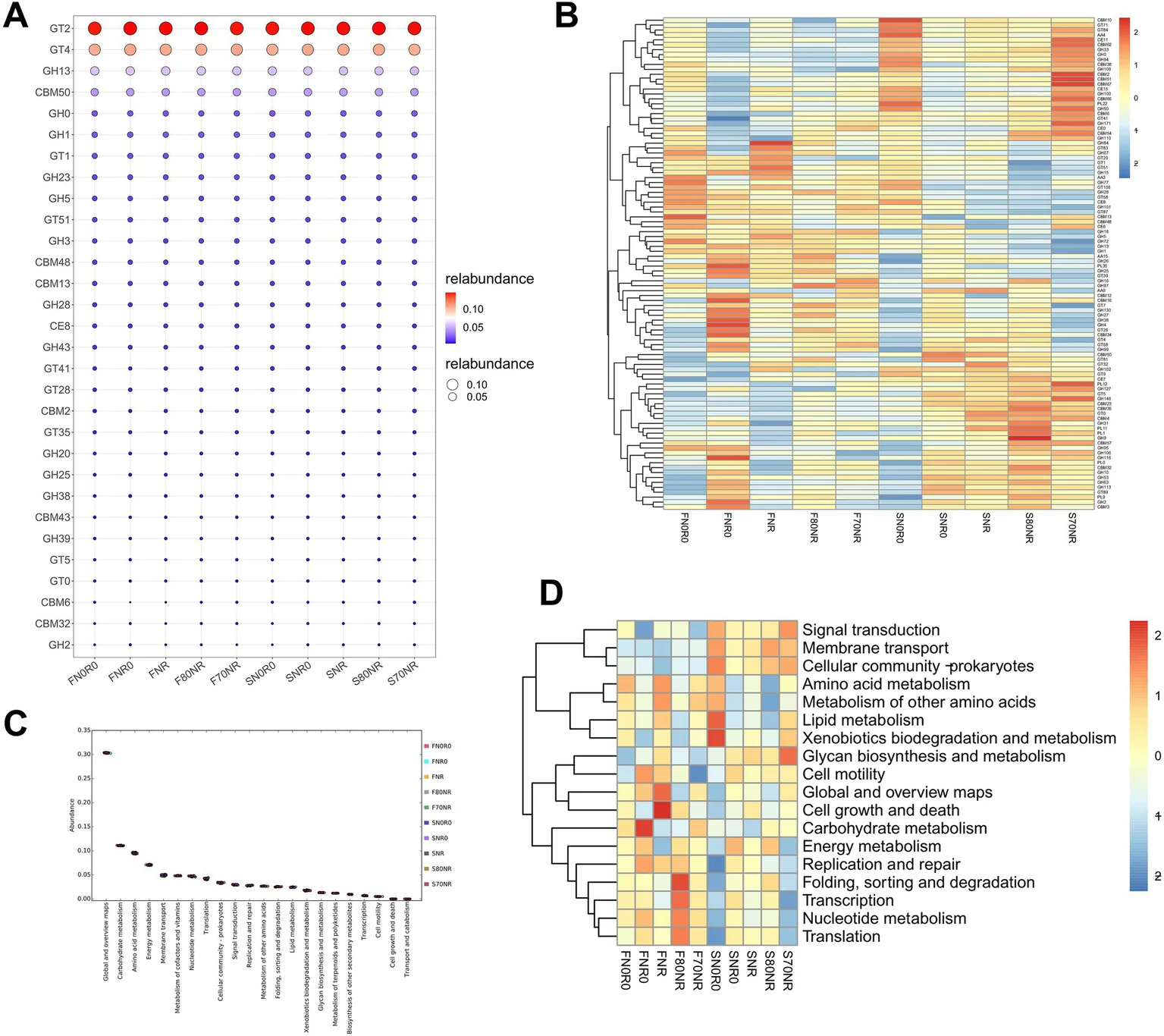
Functional profiles of carbohydrate-active enzyme (CAZy) families and KEGG pathways across fertilization regimes under continuous rice straw return. Treatments: N0R0 (no fertilizer, no straw), NR0 (conventional N without straw), NR (conventional N with straw), 80%NR (20% N reduction with straw), and 70%NR (30% N reduction with straw). Prefixes F and S denote the 0–10 cm and 10–20 cm soil layers, respectively. **(A)** Relative abundance of key CAZy families (GT, GH, CBM, CE, PL, AA) presented as bubble plots; bubble size and color intensity indicate relative abundance. **(B)** Heatmap showing the abundance distribution of CAZy families across individual samples (row *Z*-score normalized, hierarchical clustering by Ward’s method). **(C)** Relative abundance of the most abundant KEGG metabolic pathways. **(D)** Heatmap showing the abundance distribution of KEGG pathways across individual samples (row *Z*-score normalized, hierarchical clustering by Ward’s method). Color intensity in **(B,D)** indicates the *Z*-score of relative abundance (red, high; blue, low). Data are derived from metagenomic functional annotation against the CAZy and KEGG databases (*n* = 3 per treatment).

### Changes in carbon-cycling gene abundance under N reduction

Stepwise N reduction under straw return altered the abundance of carbon metabolism genes (Figure 9). coxL and cutL declined by 15.4–21.9% across both soil layers, while korA, oorA, oforA and hdrA2 increased by 5.9–18.6%. Concurrently, anaerobic carbon fixation and the reductive TCA cycle rose by 5.4–8.2%, whereas cellulolytic (bglX) and core TCA functions (mdh) remained stable (<3% variation) across all straw-return treatments. At the genus level, Bradyrhizobium and Desulfatitalea carried rTCA and 3-hydroxypropionate cycle genes under full-N conditions, with relative abundances of 0.45 and 0.12%, respectively. Under N reduction, Desulfacinum, Syntrophus, Caldanaerobacter and Azoarcus increased by 0.08–0.35%, and these genera were annotated as carrying Wood–Ljungdahl pathway and acetate-scavenging genes. The 10–20 cm layer mirrored the surface trend, with hdrA2 and korA showing 14.2 and 11.6% increases under 80%NR relative to NR.

**Figure 9:**
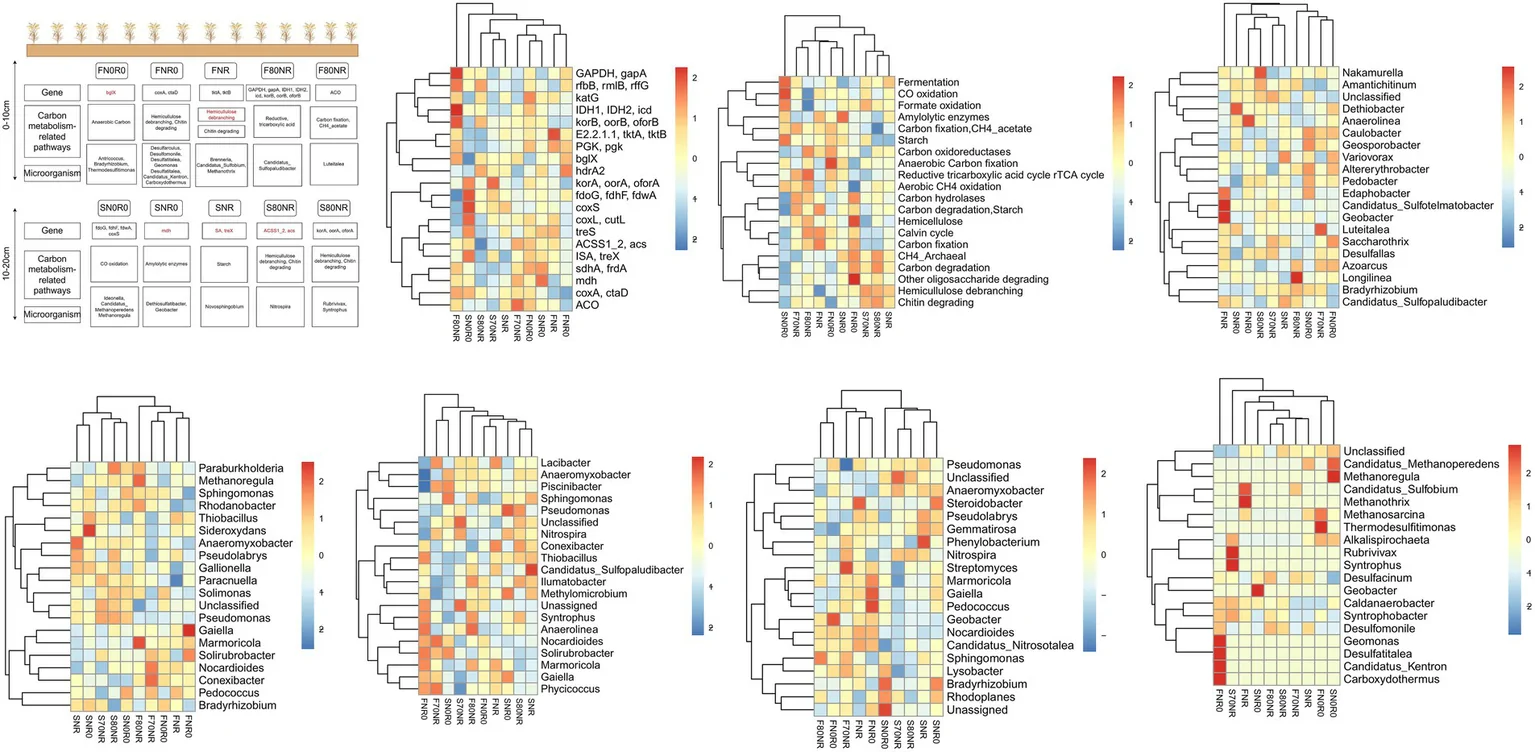
Integration of metagenomic taxonomic profiles, functional genes, and reference carbon-cycle pathways into a unified network for each treatment. Treatments: N0R0 (no fertilizer, no straw), NR0 (conventional N without straw), NR (conventional N with straw), 80%NR (20% N reduction with straw), and 70%NR (30% N reduction with straw). Prefixes F and S denote the 0–10 cm and 10–20 cm soil layers, respectively. The left panels schematically link carbon metabolism-related genes (e.g., coxA, ctaD, bglX, mdh, korA, oorA, oforA, hdrA2, ACO), metabolic pathways (CO oxidation, carbon fixation, fermentation, reductive TCA cycle, hemicellulose degrading, chitin degrading), and key microbial genera (e.g., *Nitrospira*, *Bradyrhizobium*, *Syntrophus*, *Caldanaerobacter*, *Azoarcus*, *Desulfacinum*) for each treatment. The right panels present heatmaps showing the abundance distribution (row *Z*-score normalized, hierarchical clustering by Ward’s method) of functional genes, metabolic pathways, and microbial genera across individual samples. Color intensity indicates the *Z*-score of relative abundance (red, high; blue, low). Data are derived from metagenomic sequencing and annotation against the KEGG and CAZy databases (*n* = 3 per treatment).

## Discussion

### A 20% nitrogen cut sustains yield under continuous straw return by optimizing microbial nutrient turnover

The most important agronomic finding of this study is that rice yield remained stable despite a 20% reduction in mineral nitrogen input when straw was continuously returned to the field. Although nitrogen fertilizer is traditionally regarded as the primary determinant of rice productivity, our results demonstrate that long-term straw incorporation can partially compensate for reduced mineral N inputs and sustain crop performance. This finding supports increasing evidence that crop residues should be considered not merely as organic amendments but as active regulators of nutrient cycling and soil fertility (Bossolani et al., 2023).

Several complementary mechanisms may explain the yield stability observed under 80%NR. First, straw return provides a continuous source of organic carbon and nutrients that gradually become available through microbial decomposition (Bei et al., 2022). Long-term residue incorporation promotes the accumulation of soil organic matter and increases the soil’s capacity to store and release nutrients throughout the growing season (Wu et al., 2023). Consequently, nutrient availability becomes less dependent on instantaneous fertilizer inputs and more closely linked to biologically mediated nutrient turnover. Second, the maintenance of grain yield under reduced mineral N suggests enhanced synchronization between crop N demand and soil N supply, likely mediated by improved biologically driven nutrient turnover rather than by instantaneous fertilizer availability. We acknowledge that direct measurements of plant N uptake or fertilizer N recovery were not obtained; the inference of improved N cycling efficiency is therefore based on convergent agronomic and microbial indicators (sustained yield, elevated microbial biomass, and enriched N-cycling taxa such as Nitrospira) rather than on direct isotopic or plant-tissue evidence. The maintenance of grain yield under 80%NR, alongside elevated microbial biomass and carbon management indices, is consistent with enhanced microbially mediated nutrient turnover. However, in the absence of direct plant N uptake or ^15^N recovery data, we refrain from concluding that fertilizer N use efficiency was explicitly improved. Third, enhanced microbial activity likely contributed to the sustained productivity of the system. Straw-derived carbon serves as an energy source for soil microorganisms, stimulating decomposition, nutrient mineralization, and nutrient recycling (Chen Y. et al., 2023). The increased abundance of carbon-transforming microbial groups observed under 80%NR indicates that microbial communities remained functionally active despite reduced nitrogen inputs. Such microbial regulation can maintain nutrient availability during critical growth stages and thereby buffer potential yield losses.

Interestingly, further nitrogen reduction to 70%NR did not result in substantial yield penalties, indicating that the experimental system possesses a degree of resilience to nitrogen limitation. However, evidence from aggregate stability, microbial functional diversity, and subsurface soil responses suggests that ecological trade-offs begin to emerge at this level of nitrogen reduction. Therefore, while both 20 and 30% reductions maintained grain production, the broader soil quality indicators consistently identified 20% nitrogen reduction as the more sustainable management threshold.

Meanwhile, it is noteworthy that long-term straw return with high C/N ratios has been reported to induce nitrogen immobilization and suppress soil C/N availability in some agroecosystems. However, in the present study, such negative effects were not observed under the 80%NR treatment. We attribute this to three factors. First, the 20% reduction in mineral N was implemented against a background of conventional N fertilization, ensuring that basal N availability remained sufficient to support microbial decomposition without imposing severe N limitation. Second, the continuous dual-season straw input over 9 years allowed the soil microbial community to adapt to elevated C/N conditions, as evidenced by the enrichment of N-mining taxa (Firmicutes and Actinobacteria) and sustained nitrification capacity (Nitrospira). Third, the flooded conditions during the rice season slowed straw decomposition rates, thereby moderating the intensity of microbial N competition and allowing gradual N release from both mineral fertilizer and straw mineralization to meet crop demand. These system-specific characteristics—moderate N reduction, adapted microbial communities, and water-regulated decomposition dynamics—collectively buffered the potential negative impacts of high C/N straw on soil N availability.

### Moderate nitrogen reduction improved carbon quality rather than simply increasing SOC quantity

A notable outcome of this study is that moderate nitrogen reduction primarily enhanced the quality and biological activity of soil carbon rather than substantially increasing total SOC storage. Although total organic carbon varied relatively little among straw-return treatments, significant increases were observed in readily oxidizable organic carbon, water-soluble organic carbon, microbial biomass carbon, and carbon management indices under 80%NR. These results indicate that the effects of nitrogen reduction are better reflected by changes in carbon functionality than by changes in bulk carbon stocks alone. In contrast, labile carbon fractions respond rapidly to changes in substrate supply, microbial activity, and nutrient availability. The pronounced enrichment of ROOC, WSOC, and MBC therefore suggests that moderate nitrogen reduction accelerated the transformation of straw-derived carbon into biologically active pools. One possible explanation is that moderate nitrogen limitation increased microbial carbon-use efficiency (Wu et al., 2025). Under conditions of excessive nitrogen availability, microbial communities may allocate a larger proportion of assimilated carbon toward respiration rather than biomass production (He et al., 2025). By slightly reducing nitrogen availability while maintaining substantial carbon inputs through straw return, the system may have shifted toward more efficient microbial utilization of carbon substrates. The observed increase in microbial biomass carbon supports this interpretation.

The enhancement of labile carbon fractions is particularly important because these pools serve as precursors for long-term SOC stabilization. According to the microbial carbon pump framework, microbial processing converts plant-derived organic matter into microbial residues and transformed organic compounds that contribute disproportionately to persistent soil carbon (Craig et al., 2022). Therefore, the increase in active carbon fractions observed under 80%NR should not be interpreted as evidence of reduced carbon stability. Instead, it may represent an early stage in the formation of more stable carbon pools. The higher carbon management index under moderate nitrogen reduction further supports this interpretation. CMI integrates both carbon quantity and carbon lability and is widely regarded as an indicator of soil quality and carbon sequestration potential. The superior performance of 80%NR suggests that moderate nitrogen reduction optimized the balance between carbon input, microbial processing, and carbon retention.

By contrast, the additional reduction in nitrogen under 70%NR did not produce further improvements in carbon quality indicators. This pattern suggests the existence of a threshold response. Once nitrogen availability becomes too limited, microbial decomposition and nutrient cycling may become constrained, reducing the efficiency with which straw-derived carbon is transformed and stabilized. Consequently, our results support the concept that moderate rather than severe nitrogen reduction maximises the beneficial effects of straw return on soil carbon dynamics (Bei et al., 2022).

### Aggregate protection and chemical recalcitrance jointly enhanced SOC persistence

Beyond increasing biologically active carbon pools, moderate nitrogen reduction also promoted mechanisms associated with long-term carbon stabilization. Our results indicate that SOC persistence under straw return was regulated through the combined action of aggregate-mediated physical protection and shifts in organic matter chemical composition. These complementary processes provide a mechanistic explanation for why carbon accumulation was maintained despite reductions in mineral nitrogen input.

Aggregate formation represents one of the most important pathways for protecting soil organic carbon from microbial decomposition. Organic matter occluded within macroaggregates becomes physically inaccessible to decomposers and extracellular enzymes, thereby slowing carbon turnover (de Goede et al., 2025). In the present study, aggregate stability indicators remained comparable between NR and 80%NR, demonstrating that a 20% reduction in nitrogen did not weaken aggregate formation. The preferential accumulation of SOC within macroaggregate fractions further suggests that straw-derived carbon was effectively incorporated into physically protected pools. This stability likely reflects sustained microbial production of extracellular polymeric substances and residues that function as aggregate-binding agents. In contrast, evidence of declining aggregate stability emerged under 70%NR, particularly in subsurface soils. Reduced macroaggregate abundance and increased fractal dimensions suggest weakening soil structural integrity under more severe nitrogen limitation. These findings indicate that subsurface aggregates are more sensitive to nutrient constraints than surface aggregates and may therefore represent an early warning indicator of declining soil quality.

In addition to physical protection, FTIR analyses revealed important changes in the chemical composition of soil organic matter. Straw-return treatments exhibited greater relative abundance of aliphatic and aromatic functional groups, particularly under 80%NR. These compounds are generally considered more resistant to biological degradation than simple oxygen-containing carbohydrates and therefore contribute to longer residence times of soil carbon. Importantly, FTIR data should be interpreted cautiously because spectroscopic signals do not directly identify specific molecular compounds. Nevertheless, the observed enrichment of aliphatic and aromatic structures is consistent with increased chemical recalcitrance of soil organic matter. Such compositional shifts may arise from selective preservation of resistant plant-derived compounds, microbial transformation products, or both. Taken together, our results suggest that carbon stabilization under moderate nitrogen reduction is not governed by a single mechanism. Instead, long-term SOC persistence appears to emerge from the interaction between microbial processing, aggregate-mediated physical protection, and progressive enrichment of chemically resistant organic matter. This integrated stabilization pathway provides a mechanistic foundation for explaining the superior carbon sequestration performance of the 80%NR treatment.

### Microbial functional reprogramming under nitrogen reduction redirects carbon toward stabilization pathways

It is important to acknowledge that metagenomic profiling captures functional gene potential rather than actual gene expression or *in situ* metabolic flux. The mechanistic interpretations below should therefore be viewed as hypotheses supported by relative abundance data, and future transcriptomic, proteomic, or isotopic flux studies will be required to validate the proposed metabolic shifts. The integrated metagenomic evidence presented in this study reveals that moderate nitrogen reduction under continuous straw return fundamentally reprogrammed microbial carbon-cycling networks, redirecting metabolic flux from oxidative decomposition toward reductive fixation and biomass recycling pathways that favor long-term carbon stabilization. This functional transition was underpinned by coordinated shifts in community taxonomy, enzyme systems, and metabolic pathways that together constitute a depth-resilient strategy for sustaining soil carbon sequestration under reduced mineral N inputs. The enrichment of Firmicutes, Actinobacteria, and Nitrospira under 80%NR is associated with enhanced carbon stabilization, though the direct causal pathway remains to be tested. Firmicutes are renowned for their capacity to produce resistant endospores and extracellular polymeric substances (EPS), both of which contribute to the formation of stable microbial residues that constitute a major precursor pool for persistent soil organic carbon (Oliva et al., 2024; Subirats et al., 2022). The concurrent enrichment of Actinobacteria—particularly sporulating genera such as Streptomyces and Nocardioides—further supports this mechanism, as actinobacterial cell wall constituents (notably peptidoglycan and melanin-like compounds) are among the most chemically recalcitrant microbial products in soil (Yang Y. et al., 2025). Nitrospira, functioning as complete ammonia oxidizers, likely maintained nitrification capacity under reduced N availability, thereby sustaining coupled nitrification–denitrification processes that regulate soil N availability and indirectly influence microbial C-use efficiency (Wang et al., 2026). The suppression of methanogenic archaea under 80%NR is particularly significant: by reducing the competitive advantage of hydrogenotrophic methanogens, the system likely diverted electron flow away from CH₄ production and toward alternative terminal electron-accepting processes—most notably sulfate reduction and reductive acetogenesis—that generate more stable carbon intermediates.

The CAZy profiling revealed a nuanced enzymatic response that balanced the competing demands of substrate acquisition and resource conservation. The sustained abundance of GH5 (endo-cellulase) and GH43 (arabinoxylanase/β-xylosidase) under 80%NR indicates that moderate N limitation did not constrain the depolymerization of plant-derived polysaccharides, ensuring continued access to energy-rich substrates. This maintenance of cellulolytic/xylanolytic capacity is essential for straw-derived carbon entry into the microbial loop (Wang et al., 2025). However, the concurrent increase in CBM50 (chitin/peptidoglycan-binding) and chitinolytic enzymes (GH18, GH25) under deeper N reduction signals an important metabolic pivot: as mineral N became increasingly scarce, microbial communities intensified the recycling of endogenous biomass—fungal cell walls and bacterial peptidoglycan—thereby accessing alternative N sources while simultaneously generating microbial necromass. This shift from “external mining” to “internal recycling” represents a classic ecological strategy under nutrient limitation, but with profound consequences for carbon stabilization. Microbial necromass, particularly peptidoglycan and chitin residues, exhibits substantially longer residence times in soil than plant-derived polysaccharides, contributing disproportionately to the stable carbon pool (Salas et al., 2024). The progressive decline in GT1 (UDP-glycosyltransferase) under 70%NR further suggests that excessive N limitation constrained *de novo* carbohydrate biosynthesis, highlighting the threshold nature of the response: moderate reduction (80%NR) optimized the balance between substrate acquisition and biomass recycling, whereas deeper cuts (70%NR) began to impair anabolic capacity.

The carbon-cycle gene analysis revealed the most compelling evidence for metabolic reprogramming. The marked decline in coxL and cutL (CO oxidation) coupled with increased korA, oorA, and oforA (keto-acid oxidation) and hdrA2 (reductive acetyl-CoA pathway) under N reduction indicates a systematic shift from energy-yielding oxidative processes toward energy-conserving reductive pathways (Fan et al., 2025). This metabolic re-routing is linked to altered stoichiometric patterns consistent with enhanced carbon retention. Oxidative CO metabolism generates CO₂ as a terminal product, whereas reductive acetogenesis via the Wood–Ljungdahl pathway fixes CO₂ into acetate, a central precursor for microbial biomass and extracellular organic matter (Schulz-Mirbach et al., 2024). The taxonomic underpinning of this shift—replacement of oxidative autotrophs (Bradyrhizobium, Desulfatitalea) by Wood–Ljungdahl genera (Desulfacinum, Syntrophus, Caldanaerobacter) and acetate-scavenging Azoarcus—confirms that community reorganization was functionally targeted rather than stochastic. The rTCA cycle, while increasing under N reduction, was carried by different taxa than under full N, suggesting functional redundancy with altered physiological context. The stability of Calvin cycle and aerobic CH₄ oxidation genes (<3% variation) further demonstrates that core carbon-cycling infrastructure remained intact, with metabolic plasticity expressed primarily at the level of terminal electron-accepting processes and carbon fixation strategies.

The KEGG pathway analysis provided critical contextual support for this interpretation. The stability of carbohydrate metabolism and energy metabolism (<0.1‰ variation) across N-reduction treatments confirms that central catabolic capacity was preserved, while the increase in membrane transport and translation pathways indicates enhanced investment in resource acquisition and protein synthesis under N limitation. This pattern—maintained core metabolism with upregulated acquisition machinery—is consistent with the ecological strategy of “lean” resource economies under nutrient stress, where microorganisms maximize substrate capture efficiency rather than growth yield (Wang et al., 2023; Duan et al., 2025). The progressive decline in amino-acid metabolism under deeper N reduction (70%NR) suggests, however, that this strategy has limits: once N availability falls below a critical threshold, protein synthesis becomes constrained, potentially explaining the subsoil-specific decline in microbial functional diversity observed at this treatment level.

### An integrated hypothesized mechanistic framework

Synthesizing these observations, we propose an integrated conceptual framework in which moderate N reduction under continuous straw return is consistently associated with three interconnected processes (Figure 10). First, taxonomic reorganization enriches spore-forming and EPS-producing taxa that generate chemically recalcitrant microbial residues. Second, enzymatic reprogramming sustains plant-polysaccharide degradation while intensifying endogenous biomass recycling, thereby shifting the carbon balance from labile plant inputs toward more stable microbial necromass. Third, metabolic pathway reconfiguration redirects electron flow from oxidative CO loss toward reductive CO₂ fixation via the Wood–Ljungdahl pathway, generating acetate that serves as both a carbon sink and trophic link to syntrophic communities (Shi et al., 2025). The net effect is enhanced carbon persistence through the combined action of microbial carbon pump inputs, aggregate-physical protection (as evidenced by maintained macroaggregate stability under 80%NR), and chemical recalcitrance of aliphatic/aromatic structures identified by FTIR. The threshold response—where 70%NR undermined subsoil aggregate stability and reduced functional diversity—highlights that this stabilizing state is not infinitely scalable. Excessive N limitation likely constrained the production of extracellular binding agents necessary for aggregate formation, while also limiting protein synthesis capacity required for maintaining diverse metabolic functions (Balakrishnan and Cremer, 2023).

**Figure 10:**
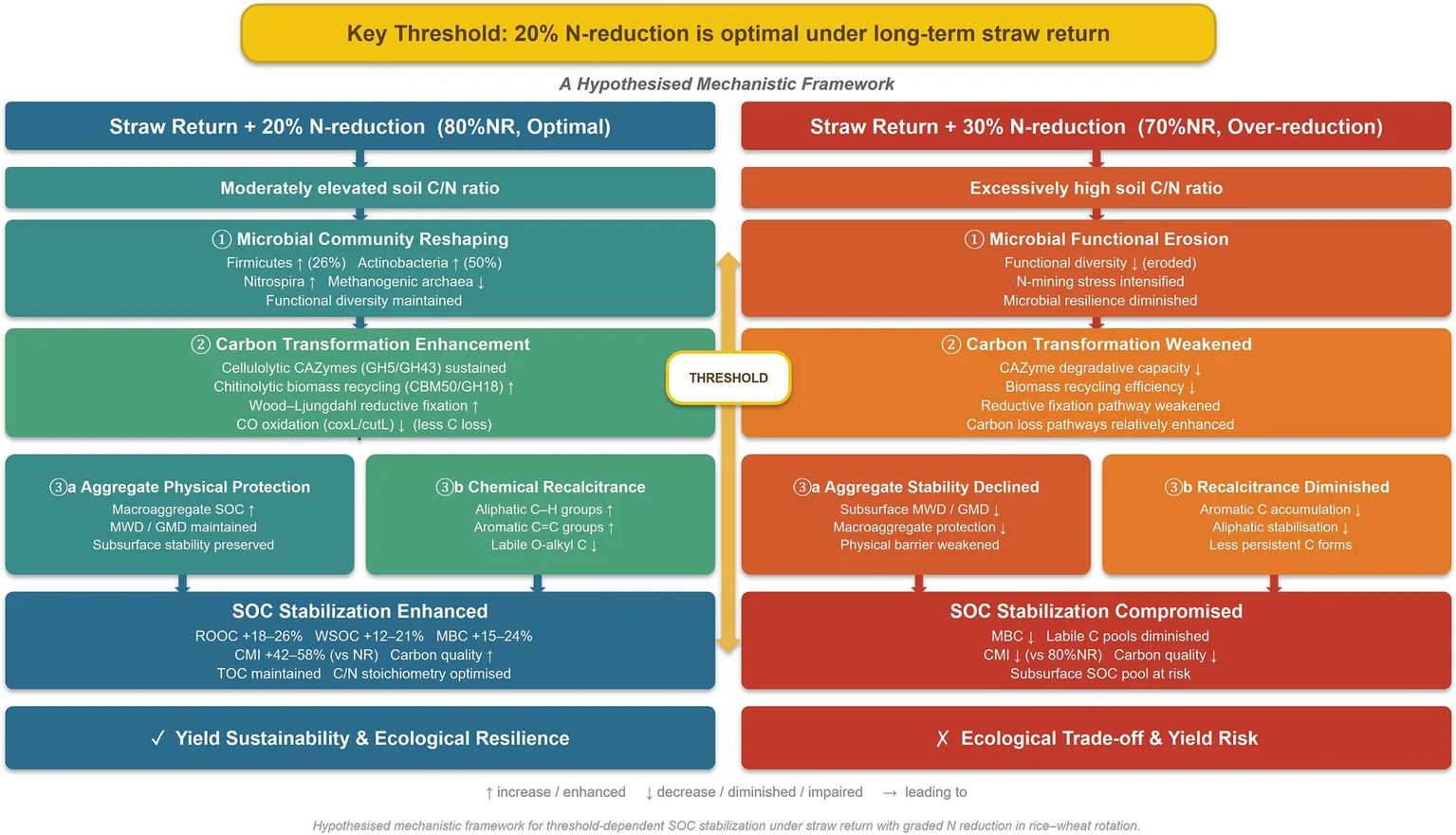
Hypothesized conceptual framework of soil organic carbon stabilization under straw return with 20% nitrogen reduction. Elevated soil C/N under 80%NR selects for carbon-stabilizing taxa (Firmicutes, Actinobacteria, Nitrospira, methanogens), reprograms enzymes toward sustained cellulolysis (GH5/GH43) and intensified biomass recycling (CBM50/GH18/GH25), and redirects carbon fixation from CO oxidation to reductive pathways (Wood–Ljungdahl, rTCA). These processes converge on aggregate-mediated physical protection and chemical recalcitrance, maximizing carbon management index while maintaining yield. The 20% reduction represents an optimal threshold; deeper cuts (70%NR) trigger subsoil functional decline. Note that the “20% threshold” refers to the optimal N-reduction level within the tested gradients (0, 20, and 30%) and does not imply a precisely quantified ecological tipping point.

### Study limitations and future perspectives

Several limitations of this study should be acknowledged. First, the N-reduction gradient included only two levels (20 and 30%), and finer gradients (e.g., 10, 15, 25%) would be needed to pinpoint the exact threshold position; our findings should therefore be interpreted as identifying an optimal range within the tested gradients rather than a precise ecological tipping point. Second, although this long-term field experiment has been continuously operated for 9 years and can mitigate random disturbances induced by interannual climatic fluctuations, sequential multi-season sampling is still required in future research to clarify the seasonal and interannual evolution characteristics of relevant indicators. Third, metagenomic data reflect functional gene potential rather than actual expression or metabolic flux; transcriptomic, proteomic, or stable-isotope probing would be required to validate the proposed metabolic shifts. Fourth, the mechanisms by which straw return suppresses negative C/N effects in this rice-wheat system—relative to upland systems—warrant further investigation, potentially involving isotopic tracing of straw-derived C and N. Finally, although multiple lines of evidence (carbon fractions, aggregates, FTIR, and metagenomics) converge on the 20% N-reduction threshold, direct statistical integration among these components—such as structural equation modelling—would strengthen causal inference. Future work should address these gaps through multi-year sampling, finer N gradients, and multi-omics integration with isotopic validation.

## Conclusion

Our study identifies a 20% reduction in mineral nitrogen input—within the gradients tested here under continuous straw return—as an agronomically viable and ecologically optimal strategy for sustainable rice systems. This moderate reduction maintained grain yield while simultaneously enhancing soil carbon quality and stability through three convergent mechanisms. First, it enriched labile carbon pools (ROOC, WSOC, and MBC) and elevated the carbon management index, demonstrating that carbon quality is more sensitive to nitrogen management than total carbon quantity. Second, it preserved aggregate-mediated physical protection and promoted the accumulation of chemically recalcitrant aliphatic and aromatic structures. Third, metagenomic analysis revealed a carbon-stabilising metabolic state characterized by the enrichment of Firmicutes, Actinobacteria, and Nitrospira; sustained cellulolytic capacity; intensified chitinolytic biomass recycling; and a metabolic shift from oxidative CO loss toward reductive carbon fixation via the Wood–Ljungdahl pathway. These benefits proved threshold-dependent: a 30% nitrogen reduction undermined subsurface aggregate stability and eroded microbial functional diversity. Consequently, the 20% threshold represents an optimum within the tested range, beyond which carbon-stabilizing mechanisms degrade. Our findings provide a mechanistic basis for optimizing nitrogen management in rice production systems, showing that strategic nitrogen reduction can enhance carbon functionality without compromising agronomic performance. Future research should examine whether this threshold varies with soil texture, climate, or straw quality.

## Data Availability

The datasets presented in this study can be found in online repositories. The names of the repository/repositories and accession number(s) can be found at: https://www.ncbi.nlm.nih.gov/, PRJNA1392060.

## References

[ref1] Averill C. Waring B. (2018). Nitrogen limitation of decomposition and decay: how can it occur? Glob. Chang. Biol. 24, 1417–1427. doi: 10.1111/gcb.13980, 29121419

[ref2] Balakrishnan R. Cremer J. (2023). Conditionally unutilized proteins and their profound effects on growth and adaptation across microbial species. Curr. Opin. Microbiol. 75:102366. doi: 10.1016/j.mib.2023.102366, 37625262

[ref3] Bei S. Li X. Kuyper T. W. Chadwick D. R. Zhang J. (2022). Nitrogen availability mediates the priming effect of soil organic matter by preferentially altering the straw carbon-assimilating microbial community. Sci. Total Environ. 815:152882. doi: 10.1016/j.scitotenv.2021.152882, 34998759

[ref4] Blair G. J. Lefroy R. Lisle L. (1995). Soil carbon fractions based on their degree of oxidation, and the development of a carbon management index for agricultural systems. Aust. J. Agric. Res. 46, 1459–1466. doi: 10.1071/ar9951459

[ref5] Bossolani J. W. Leite M. F. A. Momesso L. ten Berge H. Bloem J. Kuramae E. E. (2023). Nitrogen input on organic amendments alters the pattern of soil-microbe-plant co-dependence. Sci. Total Environ. 890:164347. doi: 10.1016/j.scitotenv.2023.164347, 37230351

[ref6] Chen Y. du Z. Weng Z. ( H.) Sun K. Zhang Y. Liu Q. . (2023). Formation of soil organic carbon pool is regulated by the structure of dissolved organic matter and microbial carbon pump efficacy: a decadal study comparing different carbon management strategies. Glob. Chang. Biol. 29, 5445–5459. doi: 10.1111/gcb.16865, 37424182

[ref7] Chen K. Ma T. Ding J. Yu S.’. Dai Y. He P. . (2023). Effects of straw return with nitrogen fertilizer reduction on Rice (*Oryza sativa* L.) morphology, photosynthetic capacity, yield and water–nitrogen use efficiency traits under different water regimes. Agronomy 13:133. doi: 10.3390/agronomy13010133, 30654563

[ref8] Chen S. Wang C. Zhang X. Cui J. Zhang J. Müller C. . (2026). Aligning nitrogen form with Rice preference through enhanced-efficiency fertilizers raises yield and cuts emissions. Glob. Chang. Biol. 32:e70817. doi: 10.1111/gcb.70817, 41891227

[ref9] Chen S. Zhou Y. Chen Y. Gu J. (2018). Fastp: an ultra-fast all-in-one FASTQ preprocessor. Bioinformatics 34, i884–i890. doi: 10.1093/bioinformatics/bty560, 30423086 PMC6129281

[ref10] Craig M. E. Geyer K. M. Beidler K. V. Brzostek E. R. Frey S. D. Stuart Grandy A. . (2022). Fast-decaying plant litter enhances soil carbon in temperate forests but not through microbial physiological traits. Nat. Commun. 13:1229. doi: 10.1038/s41467-022-28715-9, 35264580 PMC8907208

[ref11] de Goede S. P. C. Hannula S. E. Jansen B. Morriën E. (2025). Fungal-mediated soil aggregation as a mechanism for carbon stabilization. ISME J. 19. doi: 10.1093/ismejo/wraf074, 40249290 PMC12106282

[ref12] Duan Y. Y. Yang H. Li J. Wang N. Wu T. Sun X. D. . (2025). Ecological restoration in plateau pika-disturbed alpine meadows: soil-microbe-enzyme stoichiometry and microbial adaptations during vegetation recovery of bare patches. J. Environ. Manag. 394:127373. doi: 10.1016/j.jenvman.2025.127373, 41004966

[ref13] Fan Z. Wu H. Feng Y. Sun L. Yuan L. Muhammad T. (2025). Biochar mitigates biodegradable microplastic-induced greenhouse gas emissions in lake sediments: unraveling microbial mechanisms and particle-size effects. J. Environ. Manag. 395:127799. doi: 10.1016/j.jenvman.2025.127799, 41175489

[ref14] Gaudel G. Xing L. Shrestha S. Poudel M. Sherpa P. Raseduzzaman M. . (2024). Microbial mechanisms regulate soil organic carbon mineralization under carbon with varying levels of nitrogen addition in the above-treeline ecosystem. Sci. Total Environ. 917:170497. doi: 10.1016/j.scitotenv.2024.170497, 38301775

[ref15] Gurevich A. Saveliev V. Vyahhi N. Tesler G. (2013). QUAST: quality assessment tool for genome assemblies. Bioinformatics 29, 1072–1075. doi: 10.1093/bioinformatics/btt086, 23422339 PMC3624806

[ref16] He L. Huang X. L. Jia K. T. Wang K. H. Lan B. Gu L. . (2025). Responses of soil respiration and its temperature sensitivity to nitrogen and phosphorus depositions in a riparian zone. J. Environ. Manag. 374:124173. doi: 10.1016/j.jenvman.2025.124173, 39837141

[ref17] Kanehisa M. (2004). The KEGG resource for deciphering the genome. Nucleic Acids Res. 32, D277–D280. doi: 10.1093/nar/gkh063, 14681412 PMC308797

[ref18] Langmead B. Salzberg S. L. (2012). Fast gapped-read alignment with bowtie 2. Nat. Methods 9, 357–359. doi: 10.1038/nmeth.1923, 22388286 PMC3322381

[ref19] Li D. Liu C. M. Luo R. Sadakane K. Lam T. W. (2015). MEGAHIT: an ultra-fast single-node solution for large and complex metagenomics assembly via succinct de Bruijn graph. Bioinformatics 31, 1674–1676. doi: 10.1093/bioinformatics/btv033, 25609793

[ref20] Liu L. Greaver T. L. (2010). A global perspective on belowground carbon dynamics under nitrogen enrichment. Ecol. Lett. 13, 819–828. doi: 10.1111/j.1461-0248.2010.01482.x, 20482580

[ref21] Liu D. Song C. Xin Z. Fang C. Liu Z. Xu Y. (2023). Agricultural management strategies for balancing yield increase, carbon sequestration, and emission reduction after straw return for three major grain crops in China: a meta-analysis. J. Environ. Manag. 340:117965. doi: 10.1016/j.jenvman.2023.117965, 37121003

[ref22] Liu Y. Wang K. Liao S. Ren T. Li X. Cong R. . (2022). Differences in responses of ammonia volatilization and greenhouse gas emissions to straw return and paddy-upland rotations. Environ. Sci. Pollut. Res. Int. 29, 25296–25307. doi: 10.1007/s11356-021-17239-2, 34839441

[ref23] Nelson D. W. Sommers L. E. (1982). “Total carbon, organic carbon and organic matter,” in Methods of Soil Analysis Part 2. Chemical and Microbial Properties, eds A. L. Page, R. H. Miller, and D. R. Keeney, 539–579.

[ref24] Ni Y. Xia Z. Yang Y. Liu D. Xiang R. Ding Y. . (2024). Spatial and temporal evolution and factors influencing soil aggregate stability in the riparian zone during exposure: a case study of the water-level fluctuation zone of the three gorges reservoir, China. Sci. Total Environ. 957:177408. doi: 10.1016/j.scitotenv.2024.177408, 39522778

[ref25] Oliva R. L. Vogt C. Bublitz T. A. Camenzind T. Dyckmans J. Joergensen R. G. (2024). Galactosamine and mannosamine are integral parts of bacterial and fungal extracellular polymeric substances. ISME Commun 4:ycae038. doi: 10.1093/ismeco/ycae038, 38616925 PMC11014887

[ref26] Ren G. Zhang X. Xin X. Li M. Wang M. Yang W. . (2024). Assessing the allocations of exogenous N to the soil organic N pool in maize-wheat cropping using (15)N in situ labelling. Sci. Total Environ. 908:168397. doi: 10.1016/j.scitotenv.2023.168397, 37956836

[ref27] Salas E. Gorfer M. Bandian D. Eichorst S. A. Schmidt H. Horak J. . (2024). Reevaluation and novel insights into amino sugar and neutral sugar necromass biomarkers in archaea, bacteria, fungi, and plants. Sci. Total Environ. 906:167463. doi: 10.1016/j.scitotenv.2023.167463, 37793447

[ref28] Schulz-Mirbach H. Wichmann P. Satanowski A. Meusel H. Wu T. Nattermann M. . (2024). New-to-nature CO(2)-dependent acetyl-CoA assimilation enabled by an engineered B(12)-dependent acyl-CoA mutase. Nat. Commun. 15:10235. doi: 10.1038/s41467-024-53762-9, 39592584 PMC11599936

[ref29] Shi H. Zhang X. Liu L. Thompson F. Li X. Sun H. . (2025). Vertically stratified carbon fixation and coupling processes in deep-sea sediment. ISME Commun 5:ycaf242. doi: 10.1093/ismeco/ycaf242, 41496864 PMC12766754

[ref30] Song J. Song J. Haider S. Bai J. Yang G. Ren G. . (2025). Straw return and nitrogen-phosphorus fertilization coordinates crop yields, soil nutrient function and carbon emissions. J. Environ. Manag. 393:126950. doi: 10.1016/j.jenvman.2025.126950, 40858082

[ref31] Steinegger M. Söding J. (2017). MMseqs2 enables sensitive protein sequence searching for the analysis of massive data sets. Nat. Biotechnol. 35, 1026–1028. doi: 10.1038/nbt.3988, 29035372

[ref32] Subirats J. Sharpe H. Topp E. (2022). Fate of Clostridia and other spore-forming Firmicute bacteria during feedstock anaerobic digestion and aerobic composting. J. Environ. Manag. 309:114643. doi: 10.1016/j.jenvman.2022.114643, 35151135

[ref33] Sun Y. Wang H. Jia W. Ren K. Li B. Zou H. . (2025). Straw humification coefficient and its relation to soil carbon and nitrogen status. J. Environ. Manag. 393:127112. doi: 10.1016/j.jenvman.2025.127112, 40865318

[ref34] Vance E. D. Brookes P. C. Jenkinson D. S. (1987). An extraction method for measuring soil microbial biomass C. Soil Biol. Biochem. 19, 703–707. doi: 10.1016/0038-0717(87)90052-6

[ref35] Walkley A. J. Black I. A. (1934). An examination of the Degtjareff method for determining soil organic matter, and a proposed modification of the chromic acid titration method. Soil Sci. 37, 29–38. doi: 10.1097/00010694-193401000-00003

[ref36] Wang B. Chai Z. Chen J. Bo L. Xia Z. Cui J. . (2026). Differential responses of selectively enriched comammox Nitrospira and ammonia-oxidizing bacteria to ammonia and copper starvation under contrasting dissolved oxygen conditions. Bioresour. Technol. 455:134784. doi: 10.1016/j.biortech.2026.134784, 42106154

[ref37] Wang X. Li Y. Wang L. Duan Y. Yao B. Chen Y. . (2023). Soil extracellular enzyme stoichiometry reflects microbial metabolic limitations in different desert types of northwestern China. Sci. Total Environ. 874:162504. doi: 10.1016/j.scitotenv.2023.162504, 36863586

[ref38] Wang X. Wang L. Han X. Wang L. Zhang Y. (2025). Synthetic microbial community promotes straw Humification and improves soil organic carbon in cropland. Environ. Sci. Technol. 59, 25960–25972. doi: 10.1021/acs.est.5c07865, 41264879

[ref39] Wu M. Chen L. Chen S. Chen Y. Ma J. Zhang Y. . (2025). Soil microbial carbon and nitrogen limitation constraints soil organic carbon stability in arid and semi-arid grasslands. J. Environ. Manag. 373:123675. doi: 10.1016/j.jenvman.2024.123675, 39673853

[ref40] Wu Y. He J. Liu W. Cheng W. Shaaban M. Jiang Y. (2023). The effects of continuous straw returning strategies on SOC balance upon fresh straw incorporation. Environ. Res. 232:116225. doi: 10.1016/j.envres.2023.116225, 37247652

[ref41] Xin J. Yan L. Cai H. (2024). Response of soil organic carbon to straw return in farmland soil in China: a meta-analysis. J. Environ. Manag. 359:121051. doi: 10.1016/j.jenvman.2024.121051, 38723507

[ref42] Yang Y. Gunina A. Chen J. Wang B. Cheng H. Wang Y. . (2025). Unfolding the potential of soil microbial community diversity for accumulation of Necromass carbon at large scale. Glob. Chang. Biol. 31:e70292. doi: 10.1111/gcb.70292, 40503616

[ref43] Yang J. Lin P. Liu Q. Xia Y. Zhao W. Wang D. . (2026). From major crops to sugarcane: rethinking nitrogen use efficiency for a sustainable future. Plant Physiol. Biochem. 232:111170. doi: 10.1016/j.plaphy.2026.111170, 41759429

[ref44] Yang H. Zou Q. Zhang J. Xia Q. Ten X. Huang X. . (2025). Metagenomic reconstruction of microbial structure and carbon cycling for annual crop productivity: influence of long-term straw mulching and nitrogen. Appl. Soil Ecol. 211:106146. doi: 10.1016/j.apsoil.2025.106146

[ref45] Zhang J. Zhao S. Liu Y. Liang H. Wang T. Zhao Y. . (2023). Differences in methane and nitrous oxide emissions and soil bacteria communities between straw return methods in Central China. Environ. Sci. Pollut. Res. Int. 30, 1167–1175. doi: 10.1007/s11356-022-21883-7, 35915302

[ref46] Zhu W. Lomsadze A. Borodovsky M. (2010). Ab initio gene identification in metagenomic sequences. Nucleic Acids Res. 38:e132. doi: 10.1093/nar/gkq275, 20403810 PMC2896542

[ref47] Zhu C. Zhong W. Han C. Deng H. Jiang Y. (2023). Driving factors of soil organic carbon sequestration under straw returning across China's uplands. J. Environ. Manag. 335:117590. doi: 10.1016/j.jenvman.2023.117590, 36863145

