# Supplementary material for: A 20% nitrogen reduction threshold optimizes soil carbon stability and microbial functional resilience under long-term straw return in a rice–wheat rotation
Source: Front Microbiol. 2026 Sep 14;17:1928083. doi: 10.3389/fmicb.2026.1928083 (PMC13649076; doi:10.3389/fmicb.2026.1928083)
Supplement: Supplementary file 1 [file Table_1.DOCX]

| Group | Treatment Description | Mineral N (kg/ha) | Wheat Straw Return |
| --- | --- | --- | --- |
| N0R0 | No fertilizer, no straw | 0 | No |
| NR0 | 100% mineral N, no straw | 210 | No |
| NR | 100% mineral N + full straw return | 210 | Yes (full) |
| 80%NR | 80% mineral N + full straw return | 168 | Yes (full) |
| 70%NR | 70% mineral N + full straw return | 147 | Yes (full) |

**Experimental treatments and nitrogen application rates during the rice-growing season**

**Fertilizer type, application rate and straw return amount under different treatments (unit: g 30 m⁻²)**

| Treatment | Wheat season | | | | Rice season | | | |
| --- | --- | --- | --- | --- | --- | --- | --- | --- |
|  | Urea(46% N) | Calcium superphosphate(12% P₂O₅) | Potassium chloride(60% K₂O) | Returned wheat straw | Urea(46% N) | Calcium superphosphate(12% P₂O₅) | Potassium chloride(60% K₂O) | Returned rice straw |
| N0R0 | 0 | 0 | 0 | 0 | 0 | 0 | 0 | 0 |
| NR0 | 1174 | 2249 | 540 | 0 | 1369 | 2624 | 840 | 0 |
| NR | 1174 | 2249 | 540 | 18 | 1369 | 2624 | 840 | 30 |
| 80%NR | 939 | 2249 | 540 | 18 | 1095 | 2624 | 840 | 30 |
| 70%NR | 821 | 2249 | 540 | 18 | 959 | 2264 | 840 | 30 |
