# Supplementary material for: A 20% nitrogen reduction threshold optimizes soil carbon stability and microbial functional resilience under long-term straw return in a rice–wheat rotation
Source: Front Microbiol. 2026 Sep 14;17:1928083. doi: 10.3389/fmicb.2026.1928083 (PMC13649076; doi:10.3389/fmicb.2026.1928083)
Supplement: Supplementary file 2 [file Table_2.DOCX]

**Summary statistics of gene sequences**

| Sample ID | Gene number | Total length(bp) | Average length(bp) | Max length(bp) | Min length(bp) |
| --- | --- | --- | --- | --- | --- |
| FN0R0-1 | 1136748 | 4.27E+08 | 375 | 6873 | 102 |
| FN0R0-2 | 1088132 | 4.12E+08 | 378 | 7197 | 102 |
| FN0R0-3 | 1114615 | 4.18E+08 | 375 | 7368 | 102 |
| FNR0-1 | 1194393 | 4.53E+08 | 379 | 8679 | 102 |
| FNR0-2 | 1143479 | 4.33E+08 | 378 | 5829 | 102 |
| FNR0-3 | 1155916 | 4.41E+08 | 381 | 8679 | 102 |
| FNR-1 | 1210821 | 4.57E+08 | 377 | 6489 | 102 |
| FNR-2 | 1163247 | 4.4E+08 | 378 | 5661 | 102 |
| FNR-3 | 1166434 | 4.44E+08 | 380 | 8679 | 102 |
| F80NR-1 | 1043852 | 4.06E+08 | 388 | 8679 | 102 |
| F80NR-2 | 1011105 | 3.92E+08 | 387 | 8679 | 102 |
| F80NR-3 | 1055114 | 4.08E+08 | 386 | 8484 | 102 |
| F70NR-1 | 1016719 | 3.95E+08 | 388 | 8679 | 102 |
| F70NR-2 | 1163242 | 4.44E+08 | 381 | 8679 | 102 |
| F70NR-3 | 1049400 | 3.98E+08 | 379 | 6237 | 102 |
| SN0R0-1 | 1141567 | 4.36E+08 | 382 | 9399 | 102 |
| SN0R0-2 | 1078118 | 4.09E+08 | 379 | 5610 | 102 |
| SN0R0-3 | 1128198 | 4.31E+08 | 381 | 5685 | 102 |
| SNR0-1 | 1009959 | 3.86E+08 | 382 | 6315 | 102 |
| SNR0-2 | 1040463 | 4.05E+08 | 389 | 6936 | 102 |
| SNR0-3 | 1045764 | 4.07E+08 | 389 | 8679 | 102 |
| SNR-1 | 1006575 | 3.94E+08 | 391 | 8679 | 102 |
| SNR-2 | 1216381 | 4.63E+08 | 380 | 6315 | 102 |
| SNR-3 | 1163247 | 4.47E+08 | 383 | 8679 | 102 |
| S80NR-1 | 1061900 | 4.14E+08 | 389 | 8679 | 102 |
| S80NR-2 | 1095125 | 4.29E+08 | 392 | 6372 | 102 |
| S80NR-3 | 1225170 | 4.73E+08 | 385 | 8679 | 102 |
| S70NR-2 | 1103031 | 4.2E+08 | 380 | 8217 | 102 |
| S70NR-3 | 1212639 | 4.63E+08 | 381 | 8679 | 102 |
| S70NR-1 | 1372804 | 5.28E+08 | 384 | 8679 | 102 |
