# Supplementary material for: A 20% nitrogen reduction threshold optimizes soil carbon stability and microbial functional resilience under long-term straw return in a rice–wheat rotation
Source: Front Microbiol. 2026 Sep 14;17:1928083. doi: 10.3389/fmicb.2026.1928083 (PMC13649076; doi:10.3389/fmicb.2026.1928083)
Supplement: Supplementary file 3 [file Table_3.DOCX]

Agronomic efficiency of nitrogen

|  | Grain yield | Nitrogen application rate | Agronomic efficiency of nitrogen |
| --- | --- | --- | --- |
| N0R0 | 7877.1 | 0 | / |
| NR0 | 10250.43 | 210 | 11.30157143 |
| NR | 10218.71 | 210 | 11.15052381 |
| 80%NR | 10024.15 | 168 | 12.78005952 |
| 70%NR | 9966.83 | 147 | 14.21585034 |

Agronomic efficiency of nitrogen (AEₙ, kg/kg) = (Grain yield in nitrogen-fertilized plot − Grain yield in zero-nitrogen plot) / Nitrogen application rate
