# Supplementary material for: A 20% nitrogen reduction threshold optimizes soil carbon stability and microbial functional resilience under long-term straw return in a rice–wheat rotation
Source: Front Microbiol. 2026 Sep 14;17:1928083. doi: 10.3389/fmicb.2026.1928083 (PMC13649076; doi:10.3389/fmicb.2026.1928083)
Supplement: Supplementary file 4 [file Table_4.DOCX]

Soil carbon-to-nitrogen (C:N) ratio at two soil depths

| soil depths | Treatments | C：N ratio |
| --- | --- | --- |
| 0-10cm | N0R0 | 8.79 |
|  | NR0 | 9.10 |
|  | NR | 9.02 |
|  | 80%NR | 9.40 |
|  | 70%NR | 9.31 |
| 10-20cm | N0R0 | 9.17 |
|  | NR0 | 9.33 |
|  | NR | 9.27 |
|  | 80%NR | 9.36 |
|  | 70%NR | 9.67 |
