# Supplementary material for: A 20% nitrogen reduction threshold optimizes soil carbon stability and microbial functional resilience under long-term straw return in a rice–wheat rotation
Source: Front Microbiol. 2026 Sep 14;17:1928083. doi: 10.3389/fmicb.2026.1928083 (PMC13649076; doi:10.3389/fmicb.2026.1928083)
Supplement: Supplementary file 5 [file Table_5.DOCX]

Variations in organic carbon functional groups of soils at different depths

| Treatment | OC functional groups (%) | | |
| --- | --- | --- | --- |
|  | O-Alkyl C (%) | Alkyl C (%) | Aromatic-C (%) |
| 0~10 cm |  | | |
| N0R0 | 10.31 | 1.12 | 1.80 |
| NR | 11.14 | 1.02 | 1.70 |
| NR0 | 11.63 | 1.52 | 2.31 |
| 80%NR | 9.83 | 1.07 | 1.98 |
| 70%NR | 10.27 | 1.45 | 1.57 |
| 10~20 cm |  | | |
| N0R0 | 9.31 | 1.16 | 1.73 |
| NR | 11.36 | 0.80 | 1.93 |
| NR0 | 10.73 | 1.20 | 2.34 |
| 80%NR | 9.96 | 1.01 | 2.20 |
| 70%NR | 9.26 | 1.06 | 1.90 |
